# Significance of Gelsolin Superfamily Genes in Diagnosis, Prognosis and Immune Microenvironment Regulation for Endometrial Cancer

**DOI:** 10.1002/cam4.70584

**Published:** 2025-02-18

**Authors:** Senwei Jiang, Minjuan Ye, Jing Wan, Qingjian Ye, Suli Qiu, Yuebo Yang, Xiaomao Li

**Affiliations:** ^1^ Department of Gynecology The Third Affiliated Hospital of Sun Yat‐Sen University Guangzhou China

**Keywords:** endometrial cancer, gelsolin superfamily, immune infiltration, immunosuppressive tumor microenvironment, immunotherapy

## Abstract

**Background:**

Previously, anti‐CTLA4 and anti‐PD‐1/PD‐L1 immunotherapies have shown limited efficacy in MSI‐H/MMR‐D endometrial cancer, leading to poor clinical outcomes. The gelsolin superfamily, which includes *GSN*, *SCIN*, *VILL*, *VIL1*, *CAPG*, *AVIL*, *SVIL*, and *FLII*, plays crucial roles in cell motility and gene regulation.

**Aims:**

The objective of this study is to explore the potential therapeutic and prognostic implications of the gelsolin superfamily in EC.

**Materials & Methods:**

Data from TCGA, GEPIA, THPA, UALCAN, and Kaplan–Meier plotter databases were analyzed to investigate the expression and clinical relevance of gelsolin superfamily members. Co‐expression networks of the gelsolin superfamily were assessed using LinkedOmics, GeneMANIA, and NetworkAnalyst. The relationship between gelsolin superfamily and immune cell infiltration was investigated using TIMER, ImmuCellAI, and GEPIA.

**Results:**

We found that high expressions of *CAPG*, *AVIL*, and *SVIL* were associated with poor prognosis, while high expressions of *GSN* and *FLII* were linked to better outcomes in EC. Functional enrichment analysis indicated the involvement of gelsolin superfamily members in pathways related to estrogen response, MYC targets, epithelial–mesenchymal transition, TGF beta signaling, MTORC1 signaling, oxidative phosphorylation, inflammatory response, and IL6‐JAK‐STAT3 signaling. Furthermore, gelsolin superfamily members demonstrated strong correlations with the levels of monocytes, natural killer T, naive CD4+ T, follicular helper T, and central memory T in EC. In vitro studies showed that silencing CAPG and FLII could inhibit proliferation and metastasis in endometrial cancer cell lines.

**Conclusion:**

These findings indicate the significant association of gelsolin superfamily members with prognosis and immunological status in endometrial cancer.

## 1 Introduction

Endometrial cancer (EC), also known as uterine corpus endometrial carcinoma, ranks as the second most prevalent gynecologic malignancy worldwide [1]. Routine treatments such as surgery, radiotherapy, chemotherapy, and hormonal therapy have achieved a 5‐year survival rate of over 95% for early‐stage patients with localized tumors [2]. However, prognosis deteriorates significantly for advanced cases, with survival rates dropping to 68% for regional spread and 17% for metastatic disease [3]. The limited efficacy of traditional therapies in managing advanced EC underscores the urgent need for innovative and more effective therapies [4].

EC is typically classified into two main subtypes: type I, which includes endometrioid adenocarcinoma, and type II, which encompasses serous, clear cell, and undifferentiated carcinomas [3]. Recent analyses of cancer genome atlas data have identified four distinct molecular subgroups within endometrioid and serous EC, signaling a shift toward targeted molecular therapies and immunotherapies [5]. Besides these four subgroups, understanding the prognostic significance of novel factors and molecular targets could improve each patient's tailored treatment [6]. Immunotherapies targeting immune checkpoint proteins like cytotoxic T lymphocyte‐associated‐antigen 4 (CTLA4) and programmed death‐1/ligand‐1(PD‐1/PD‐L1) have shown promise in various cancers but have only shown partial effectiveness in EC with specific molecular features [7]. The tumor microenvironment's immunosuppressive nature plays a role in limiting the efficacy of immunotherapies in EC, necessitating a deeper understanding of immune‐related genes and immune cell infiltration [8].

The gelsolin superfamily, comprising seven distinct proteins highly conserved across species, plays a pivotal role in actin filament remodeling and diverse cellular processes. This family includes gelsolin (gene: *GSN*), adseverin (gene: *SCIN*), villin (gene: *VILL* and *VIL1*), capG (gene: *CAPG*), advillin (gene: *AVIL*), supervillin (gene: *SVIL*), and flightless I (gene: *FLII*). All of these proteins contain three or six homologous repeats of a structural unit known as the gelsolin‐like (G) domain. They play distinct and nonoverlapping roles in various cellular processes, such as actin filament reorganization, cellular movement regulation, phagocytosis modulation, apoptosis regulation, and control of genetic expression [9]. Further evidence indicates that, despite the key role of gelsolin superfamily members in controlling cellular processes, they can function as immunoregulatory genes in various human malignancies [10]. However, while the functions of gelsolin superfamily members have been extensively studied, their potential prognostic and immunoregulatory impacts on EC remain to be thoroughly evaluated.

To address this knowledge gap, we aimed to investigate the expression of gelsolin superfamily members and their predictive relevance for EC patients' survival. We intended to reveal the molecular biological functions of gelsolin superfamily genes using multidimensional database analysis, therefore offering insight into a new mechanism implicated in EC carcinogenesis and cancer immunology. By shedding light on the activities of the gelsolin superfamily in EC, our findings may offer valuable insights into tumor biology and immune responses in EC patients.

## 2 Materials and Methods

### 2.1 Data Collection

The Cancer Genome Atlas (TCGA) database was utilized to acquire mRNA expression data and clinical information for TCGA‐UCEC. The following samples were excluded: (1) repeated sequencing results; (2) insufficient survival information; (3) insufficient clinical data (age, gender, location of primary tumor, metastatic status). This study specifically focused on a cohort of 548 patients diagnosed with endometrial cancer, for whom extensive clinical data was available, such as age, gender, location of primary tumor, metastatic status, and death time. The gene expression profiles were derived from high‐throughput RNA sequencing of endometrial cancer tissue samples. The raw data underwent processing to convert into Fragments Per Kilobase of Transcript per Million Mapping Reads (FPKM) for subsequent analysis [11].

### 2.2 Online Database

The Gene Expression Profiling Interactive Analysis (GEPIA) (http://gepia2021.cancer‐pku.cn/) was used to create a comparison map between cancer and normal samples based on the expression levels of members belonging to the gelsolin superfamily across 33 different types of cancer [12]. Furthermore, using TCGA‐UCEC data, gene expression correlation coefficients were calculated using Pearson and Spearman methodologies.

The Human Protein Atlas (HPA) was employed to analyze the protein expression of gelsolin superfamily members in both normal endometrium (3 samples) and endometrial cancer (10 samples) [13].

The CCLE dataset, accessible at https://www.broadinstitute.org/ccle, is commonly used to characterize the genetic features of cancer cells [14]. We used the CCLE database to investigate gelsolin superfamily gene expression in endometrial cancer cell lines. Log2‐transformed expression values were plotted using the Depmap Portal database.

UALCAN (http://ualcan.path.uab.edu) was used to explore the connection between gelsolin superfamily gene expression, total protein expression, phosphoprotein expression, and clinical features of endometrial cancer patients, using the t‐test to assess differences in gene expression levels [15].

This research employed the Kaplan–Meier plotter (http://kmplot.com/analysis/) to explore the correlation between expression levels of genes in the gelsolin superfamily and the prognosis of patients with endometrial cancer [16]. The patient cohorts were determined using the automatic best cutoff selection method. A total of 542 patients were included in the analysis of overall survival, while 422 patients were included in the analysis of progression‐free survival.

The study utilized cBioPortal (http://www.cbioportal.org) to analyze genetic alterations in gelsolin superfamily genes in endometrial cancer (EC). Through cBioPortal, the researchers accessed mutation data, copy number alterations, protein levels, mRNA expression changes, and DNA methylation values related to the gelsolin superfamily genes in EC.

GeneMANIA (http://genemania.org/) was utilized to establish a gene–gene interaction (GGI) network based on co‐expression genes of the gelsolin superfamily, considering physical relationships, protein domains, co‐localization, pathways, and genetic interactions [17].

NetworkAnalyst 3.0 (https://www.networkanalyst.ca/) was utilized to generate tailor‐made protein–protein interaction (PPI) networks specific to the uterus, TF–gene interactions, and protein–chemical interactions of the gelsolin superfamily genes [18]. This detailed analysis offered insights into the protein–protein interactions, regulatory pathways, and chemical associations involving the gelsolin superfamily genes within the context of EC.

Enrichr (https://maayanlab.cloud/Enrichr/) was used for conducting gene ontology (GO) and Kyoto Encyclopedia of Genes and Genomes (KEGG) analyses to identify closely associated neighboring genes and relevant pathways linked to the co‐expression genes of gelsolin superfamily members [19].

GSEA (http://software.broadinstitute.org/gsea/index.jsp) software was used to categorize samples based on gene expression patterns and identify statistically significant gene sets linked to the gelsolin superfamily genes in EC [20].

TIMER, (http://timer.cistrome.org/) known as Tumor IMmune Estimation Resource, utilizes the spearman correlation modules to investigate relationships between gene expression and immune cell markers, considering factors such as tumor purity and age [21].

In our investigation, we used ImmuCellAI (http://bioinfo.life.hust.edu.cn/ImmuCellAI#!/) to examine the associations between gene expression immune cell abundance and responses to immunotherapy in the TCGA‐UCEC datasets [22].

### 2.3 Cell Culture and Cell Transfection

Cell lines Hec1A and Ishikawa were sourced from the Shanghai Institutes for Biological Sciences the Chinese Academy of Sciences and were cultured in Dulbecco's Modified Eagle Medium (Gibco) supplemented with 10% fetal bovine serum (Gibco).

Short interference RNA (siRNA) targeting CAPG and FLII were obtained from IGE BIO; the specific sequences can be found in Table S2. For transfection, 5 μg of siRNA was mixed in 300 μl of Opti‐MEM media (Life Technology), and Lipofectamine 3000 reagent (Life Technology) was used for cell transfection.

### 2.4 RNA Extraction and Quantitative Real‐Time PCR

We used Trizol reagent (Life Technology) to extract RNA from cultured cells. According to the manufacturer's protocol, reverse transcription reactions were performed with reverse transcriptase reagents (Promega). PCRs were carried out in triplicate using the Roche LightCycler 480. Primer sequences can be found in Table S2.

### 2.5 Cell Counting Kit‐8 (CCK‐8) Assays

For cell viability assessment, CCK‐8 (HUAYUN) assays were used. Cells were seeded in 96‐well plates at a density of 6 × 10^3^ cells per well, followed by the addition of 10 μL of CCK‐8 solution for a 2‐h incubation period. The optical density at 450 nm was measured for each well.

### 2.6 Wound Healing and Migration Assays

An assay for wound healing was performed by growing cells in six‐well plates to confluence and by creating scratches with a pipette tip. We observed and documented the progress of cell migration 24 h after wounding. Transwell chambers (BD) were used for migration assays. The lower chamber was chemoattracted with 10% fetal bovine serum medium. Cells were stained with crystal violet after 24 h of migration and then photographed.

### 2.7 Statistical Analysis

Statistical analysis was conducted using box plots to assess gelsolin superfamily gene expression in EC patients. The ROC curve and Youden Index were employed to determine the gene expression cutoff value. An analysis of gene expression and clinical characteristics was conducted using the Wilcoxon signed‐rank test and logistic regression. Kaplan–Meier analysis was used to generate survival curves. Univariate Cox analysis was first performed to identify potential prognostic variables, followed by multivariate Cox analysis to confirm prognostic factors. SPSS software (version 24.0) and R (version 3.6.4) were used for statistical analyses. Data visualization was performed with GraphPad Prism 6 and Sangerbox tools (http://www.sangerbox.com/).

## 3 Results

### 3.1 Expression Profiles of Gelsolin Superfamily Genes Across Cancer Types

We analyzed the expression patterns of gelsolin superfamily genes across various cancer types using the GEPIA database to identify differences between normal and malignant tissues.

**FIGURE 1:**
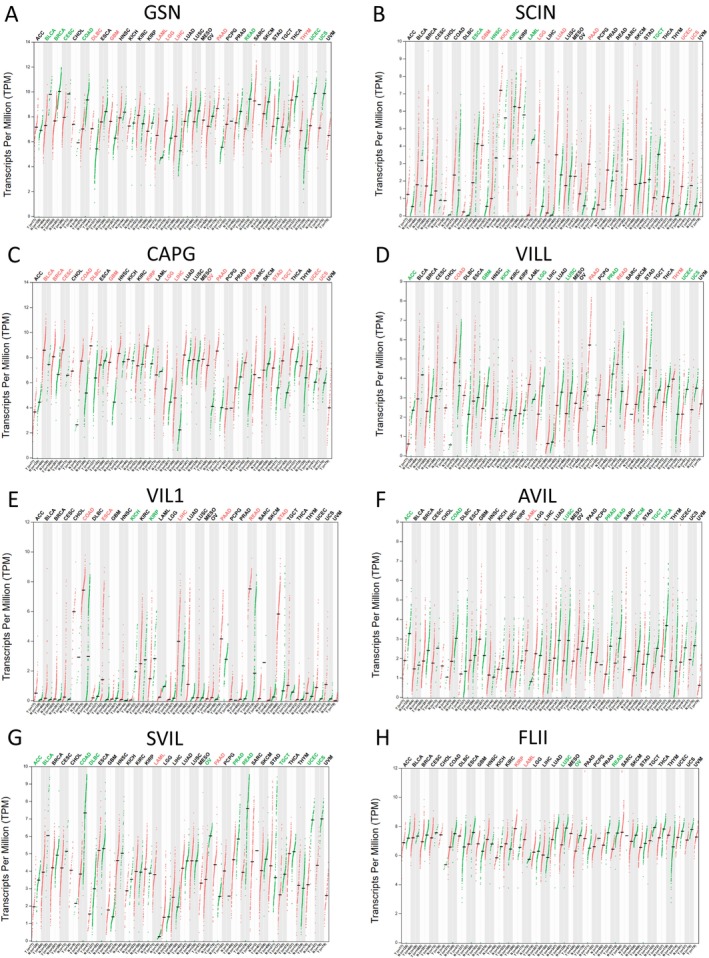
The expression levels of Gelsolin superfamily genes in UCEC. (A–H) The expression levels of GSN, SCIN, CAPG, VILL, VIL1, AVIL, SVIL, FLII in pan‐cancer.

*GSN* expression was elevated in several solid tumors, notably in DLBC, GBM, LAML, LGG, LIHC, PAAD, and THYM, whereas it decreased in BLCA, BRCA, CESC, COAD, READ, UCEC, and UCS(Figure 1A). *SCIN* expression was upregulated in GBM, KICH, LGG, LUAD, PAAD, UCEC, and UCS, but downregulated in ESCA, HNSC, KIRC, LAML, and TGCT (Figure 1B). *CAPG* RNA level was found to be significantly elevated in various solid tumors compared to normal tissues, especially in BLCA, BRCA, CESC, COAD, DLBC, GBM, KIRP, LGG, LIHC, OV, PAAD, READ, STAD, TGCT, UCEC, and UCS (Figure 1C). *VILL* expression was increased in COAD, PAAD, READ, and THYM, while reduced in ACC, GBM, KICH, LGG, LUSC, PRAD, UCEC, and UCS (Figure 1D). *VIL1* expression was raised in COAD, ESCA, LIHC, PAAD, READ, and STAD, but lowered in KICH and KIRP (Figure 1E). *AVIL* and *SVIL* expression levels were notably lower in ACC, BLCA, COAD, DLBC, LUSC, OV, PRAD, READ, SKCM, TGCT, THCA, UCEC, and UCS (Figure 1F,G). *FLII* expression was increased in KIRP and LAML, but decreased in LUSC, OV, and READ (Figure 1H).

### 3.2 Profiles of Gelsolin Superfamily Genes Expression in EC

**FIGURE 2:**
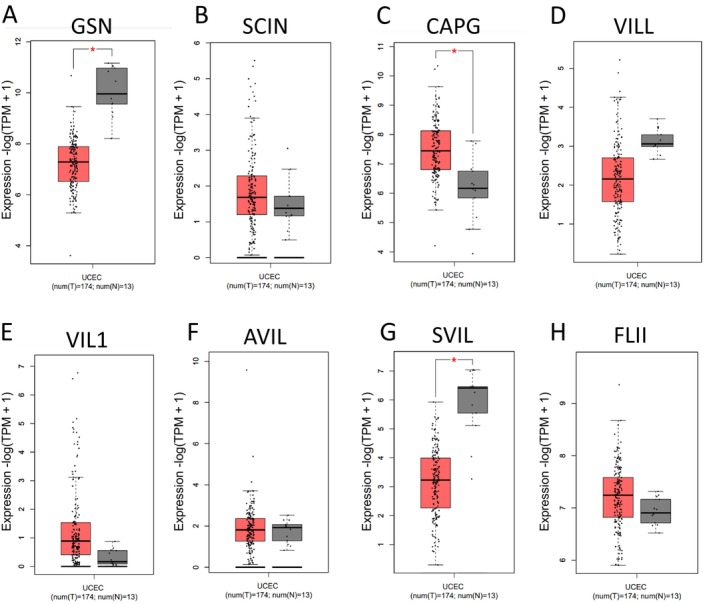
The mRNA expression levels of Gelsolin superfamily genes in 174 UCEC tumor samples and 13 paired normal ovary tissues (GEPIA) (A–H). * Indicates a statistically significant difference between the two groups.

In this study, we compared the expression of genes belonging to the gelsolin superfamily in EC samples and their corresponding normal tissues using data from the GEPIA dataset. Our analysis revealed that the mRNA expression of *CAPG* was significantly upregulated in EC tissues, while the expression levels of *GSN* and *SVIL* were downregulated in comparison to normal tissues (Figure 2). This suggests a dysregulation in the expression of genes from the gelsolin superfamily in UCEC.

**FIGURE 3:**
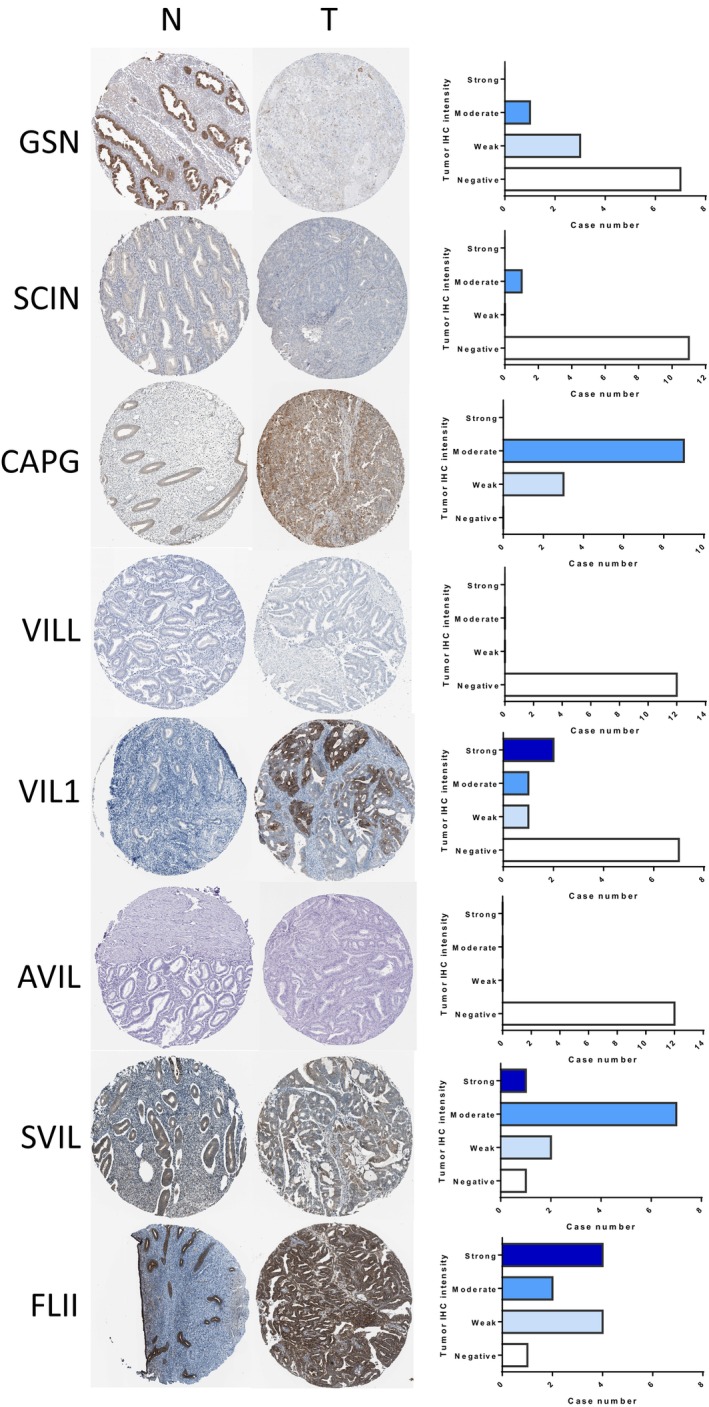
Protein expression levels of Gelsolin superfamily members in normal endometrium tissues and endometrial cancer tissues (The Human Protein Atlas). Bar charts show the IHC staining intensities of Gelsolin superfamily proteins from the endometrial cancer dictionary. N, Normal tissues; T, Tumor tissues.

Further analysis of dysregulated gelsolin superfamily members using the HPA dataset revealed that GSN, SVIL, and FLII showed strong to moderate expression levels in normal endometrial tissues, while CAPG, VIL1, SVIL, and FLII exhibited strong to moderate expression levels in EC tissues. On the other hand, SCIN, VILL, and AVIL displayed negative expression levels in both normal and malignant endometrial tissues, as depicted in Figure 3.

### 3.3 Expression Levels of Gelsolin Superfamily Genes in EC Cell Lines

**FIGURE 4:**
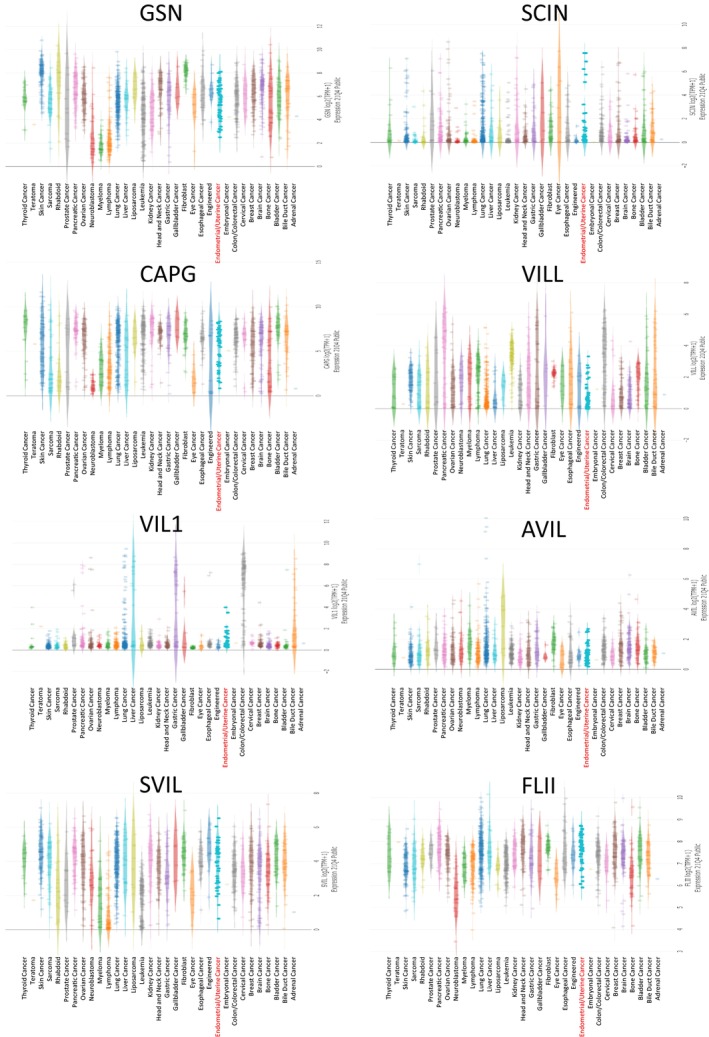
The expression of Gelsolin superfamily genes in cell lines. The number next to the lineage name represents number of cell lines in the lineage. The dashed line within a box is the mean.

Examination of gelsolin superfamily gene expression in human endometrial/uterus cancer cell lines using the Cancer Cell Line Encyclopedia (CCLE) dataset revealed moderate expression levels compared to other tumor cell lines (Figure 4).

**FIGURE 5:**
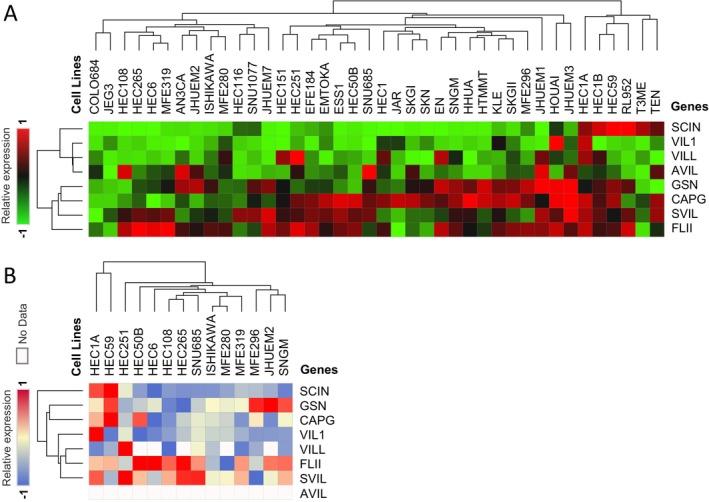
Heatmap plots representing Gelsolin superfamily genes expression levels in endomtrial/uterus cell lines acquired from the CCLE database. (A) RNA expression levels. (B) Protein expression levels.

Analysis of mRNA and protein expressions in various endometrial/uterus cell lines showed generally low mRNA expression of *SCIN*, *VIL1*, *VILL*, and *AVIL*, while *GSN*, *CAPG*, *SVIL*, and *FLII* mRNA expressions were high (Figure 5A). At the protein level, SCIN, GSN, CAPG, VIL1, and VILL were low in most endometrial/uterus cell lines, whereas FLII and SVIL exhibited high expression levels (Figure 5B).

### 3.4 Correlation Between Gelsolin Superfamily Genes Expression and Clinical Features

**FIGURE 6:**
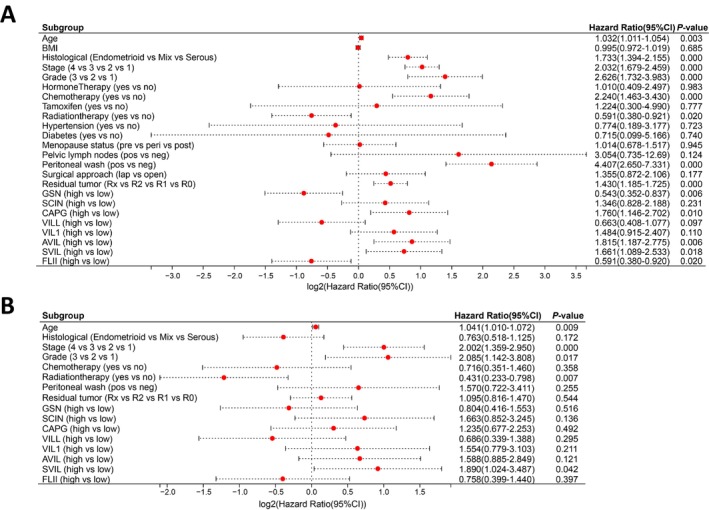
Forest plots representing correlation between Gelsolin superfamily genes expression and clinical feature. (A) OS univariate Cox analysis. (B) OS multivariate Cox analysis.

Cox regression was employed to analyze the association between the expression of gelsolin superfamily genes and clinical characteristics in the TCGA‐UCEC dataset. Univariate Cox analysis indicated that old age, histological serous cancer, advanced stage, high‐grade disease, chemotherapy, positive peritoneal washing cytology, residual tumor, and high *CAPG*, *AVIL*, and *SVIL* expressions were unfavorable predictors; however, radiation therapy and high *GSN* and *FLII* expressions were favorable predictors (Figure 6A). Multivariate Cox analysis revealed that advancing age, disease stage, utilization of radiation therapy, and the presence of *SVIL* expression were identified as independent prognostic factors for EC (Figure 6B).

**FIGURE 7:**
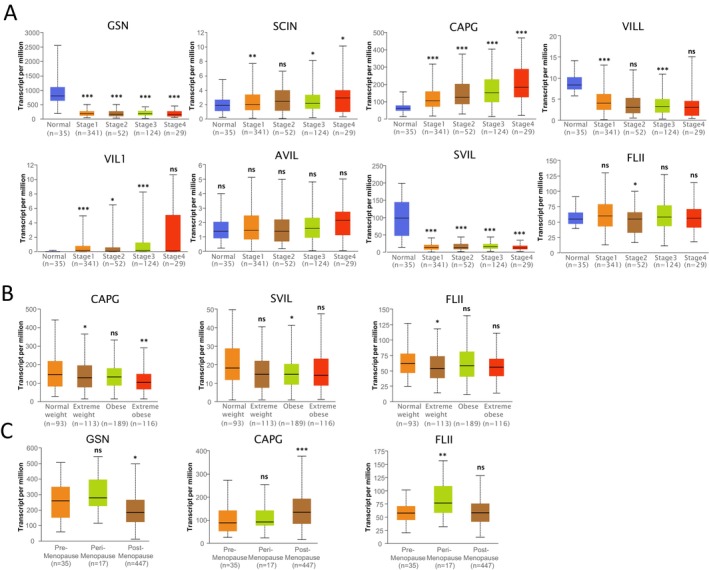
Expression of Gelsolin superfamily genes in UCEC in subgroup analyses (UALCAN). Subgroup analyses were performed based on cancer stages (A), BMI (B), and menopause status (C). ns: Not significant, *P < 0.05, **P < 0.01, ***P < 0.001.

Further examination of TCGA‐UCEC samples in UALCAN revealed that the expressions of *GSN*, *SCIN*, *CAPG*, *VILL*, *VIL1*, *SVIL*, and *FLII* were significantly altered in the stage subgroup (Figure 7A); *CAPG*, *SVIL*, and *FLII* expressions were significantly changed in the body mass index (BMI) subgroup; and *GSN*, *CAPG*, and *FLII* expressions were significantly changed in the menopause status subgroup (Figure 7B).

### 3.5 Prognostic Values of Gelsolin Superfamily Members in EC

**FIGURE 8:**
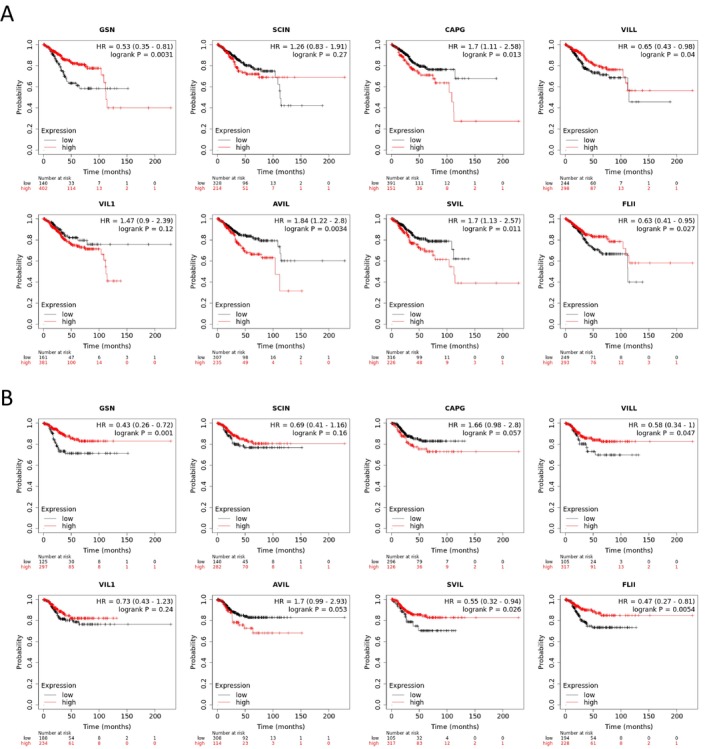
Prognostic values of transcription levels of Gelsolin superfamily members in UCEC patients in terms of overall survival (A) and relapse free survial (B). HR, hazard ratio.

We assessed the correlation between the expression levels of Gelsolin superfamily genes and survival outcomes in TCGA‐UCEC cohorts using data from the Kaplan–Meier Plotter database. Patients with low levels of *CAPG*, *AVIL*, and *SVIL* exhibited significantly longer overall survival (OS). In contrast, high expression of *GSN*, *VILL*, and *FLII* was associated with extended OS. *SCIN* and *VIL1* did not show any significant impact on OS (Figure 8A).

Furthermore, patients with high levels of *GSN*, *VILL*, and *SVIL* had significantly longer relapse‐free survival (RFS). On the other hand, *SCIN*, *CAPG*, *VIL1*, *AVIL*, and *FLII* did not demonstrate any significance in relation to RFS (Figure 8B).

### 3.6 Genetic Alterations of the Gelsolin Superfamily Genes in EC

**FIGURE 9:**
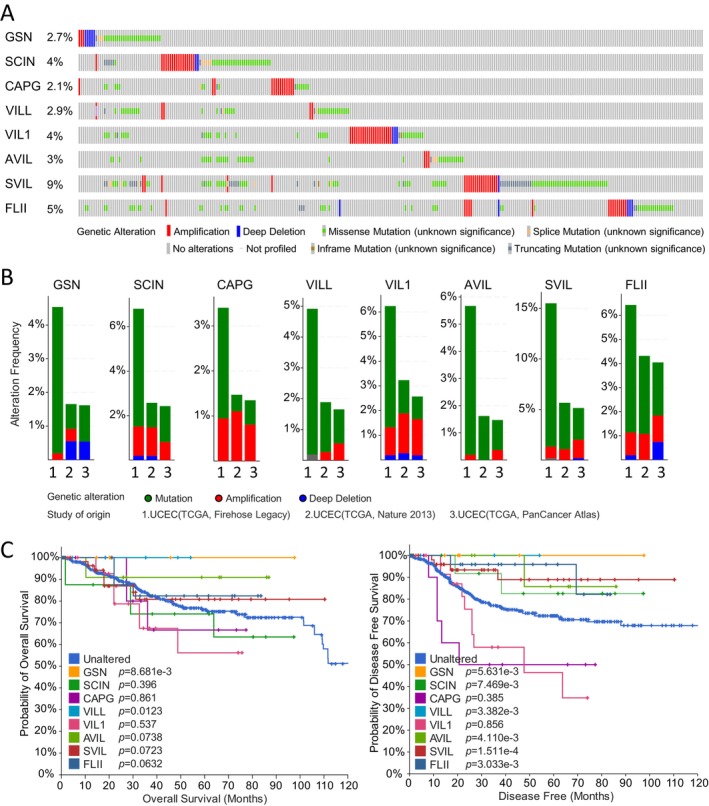
Gelsolin superfamily genes alterations in UCEC (cBioportal). (A) OncoPrint visual summary of alterations in Gelsolin superfamily genes. (B) Analysis of genetic alteration frequency in Gelsolin superfamily members in three datasets (TCGA Firehouse Legacy, TCGA Nature 2013, and TCGA PanCancer Atlas). (C) Kaplan–Meier analysis for OS and DFS in patients with or without genes alterations.

We analyzed 1450 cases of uterine corpus endometrial carcinoma from three datasets (548 from TCGA Firehose Legacy, 529 from PanCancer Atlas, and 373 from TCGA Nature 2013) in the cBioPortal database to explore the genetic alterations of the gelsolin superfamily genes in UCEC. Our investigation revealed that all eight gelsolin superfamily genes exhibited genetic alterations, ranging from 9% to 2.1% (Figure 9A). *SVIL* had the highest rate of gene modifications, whereas *CAPG* showed the lowest rate. These genes displayed amplification, mutation, and profound deletion as the three primary forms of genetic variations. Notably, mutation was the most common genetic change, particularly in *VILL*, *AVIL*, and *SVIL* (Figure 9B). Additionally, amplification was a significant contributor to alterations in *SCIN*, *VIL1*, and *SVIL*.

To assess the impact of genetic changes in each gelsolin superfamily gene on the prognosis of UCEC patients, we utilized the Kaplan–Meier method. Our results indicate that patients with mutations in *GSN* and *VILL* demonstrated a higher overall survival rate compared to those without these genetic alterations. Meanwhile, patients with mutations in *GSN*, *SCIN*, *VILL*, *AVIL*, *SVIL*, and *FLII* exhibited a more favorable prognosis (Figure 9C).

### 3.7 Identification of Genes That Were Co‐Expressed With Gelsolin Superfamily Genes in EC

**FIGURE 10:**
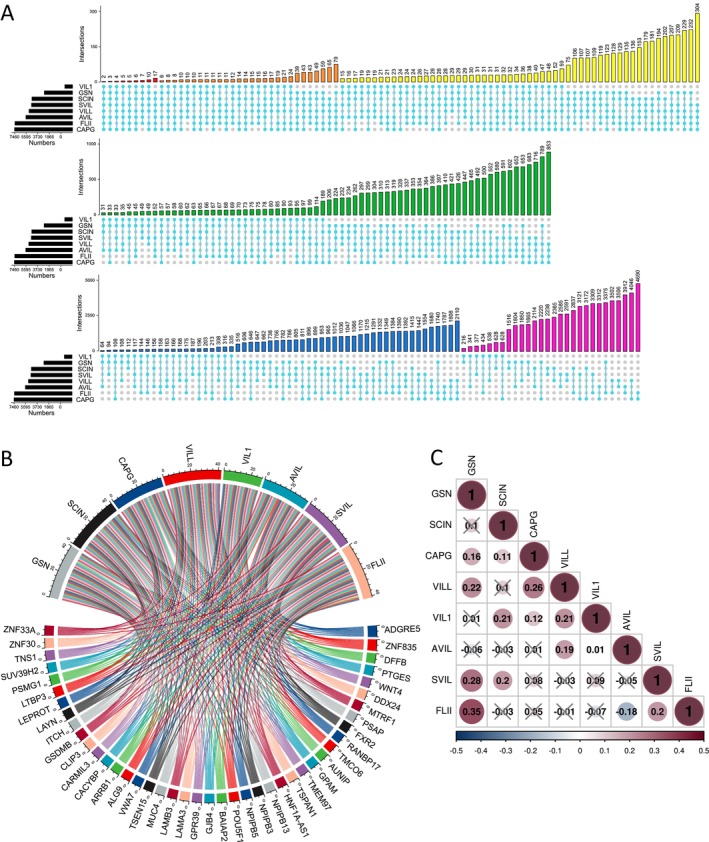
Gelsolin superfamily co‐expression genes. (A) UpSetR displayed the intersections among Gelsolin superfamily co‐expressed genes in UCEC. (B) Chord diagram showed genes co‐expressed with seven Gelsolin superfamily members. (C) Correlation between Gelsolin superfamily genes in UCEC. Insignificant correlations are indicated by crosses.

Co‐expressed genes reflect common genetic risk factors that establish functional relationships, leading us to identify a set of genes co‐expressed with gelsolin superfamily genes in UCEC using the GEPIA database. A total of 4538, 6538, 9325, 7003, 1235, 7499, 6603, and 9262 genes showed significant correlations with *GSN*, *SCIN*, *CAPG*, *VILL*, *VIL1*, *AVIL*, *SVIL*, and *FLII*, respectively (Figure 10A). Notably, two genes (*ADGRE5* and *ZNF835*) were co‐expressed with all gelsolin superfamily members. The co‐expression of other genes with the seven gelsolin superfamily members is depicted in a chord diagram in Figure 10B. Furthermore, we calculated the correlation between gelsolin superfamily genes in EC (Figure 10C).

**FIGURE 11:**
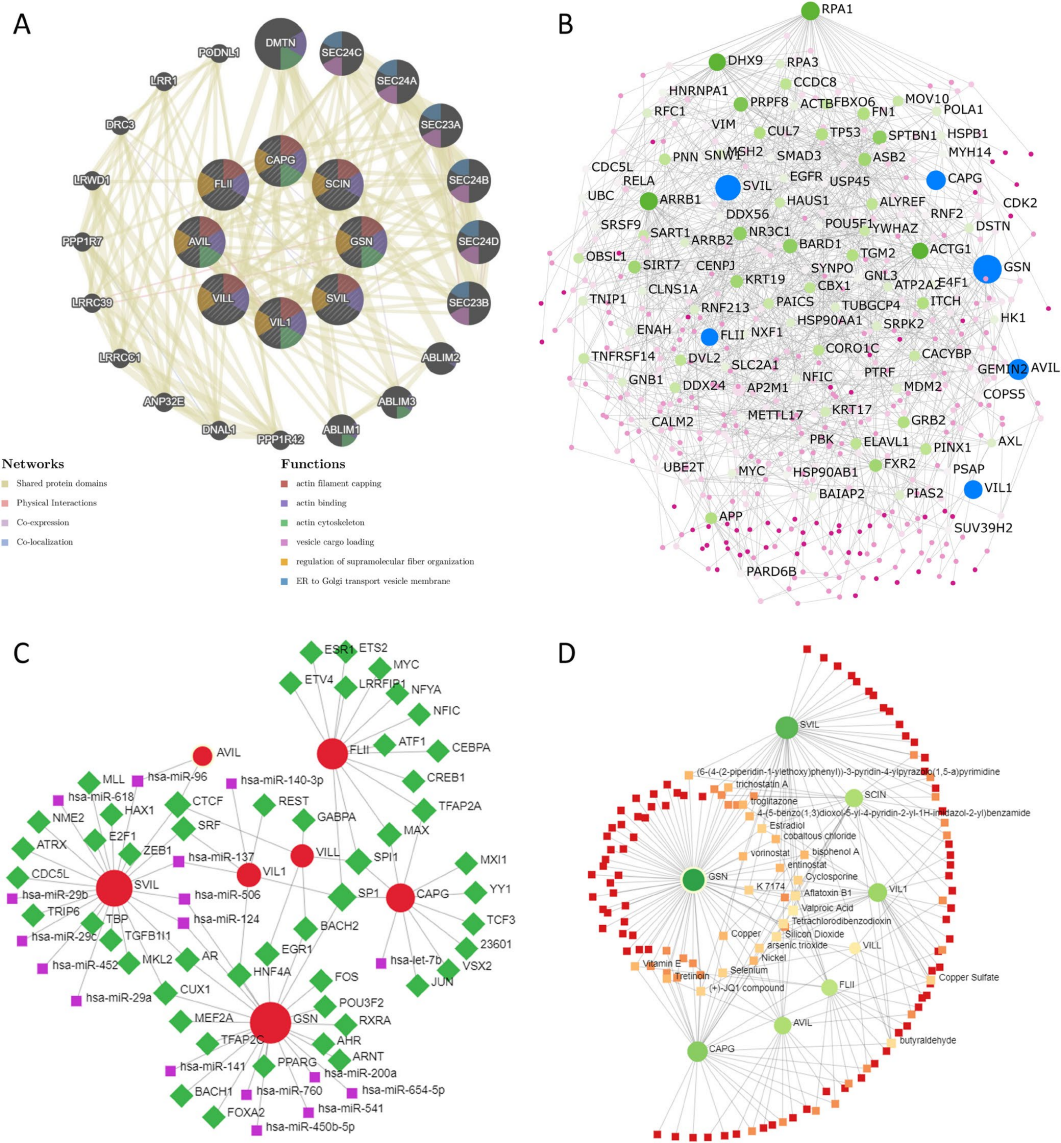
Gelsolin famliy co‐expression genes networks in EC. (A) Protein–protein interaction and function network. (B) Uterus‐specific protein–protein interaction network, (C) transcription factor‐miRNA (TF‐miRNA) coregulatory network. (D) Protein–chemical interactions network.

GeneMANIA was utilized to construct a protein co‐expressed network in the TCGA‐UCEC dataset. Notably, actin filament capping, actin binding, actin cytoskeleton, vesicle cargo loading, control of supramolecular fiber organization, and endoplasmic reticulum – Golgi transport vesicle membrane were among the top 20 substantially co‐expressed genes identified in this study (Figure 11A).

Using uterus‐specific data from the DifferentialNet database, NetworkAnalyst identified the top 5 proteins as replication protein A1 (RPA1), arrestin beta 1 (ARRB1), DExH‐box helicase 9 (DHX9), actin gamma 1 (ACTG1), and BRCA1‐associated RING domain 1 (BARD1) (Figure 11B).

Moreover, utilizing the RegNetwork database, a graph depicting TF–miRNA coregulatory interactions of gelsolin superfamily members co‐expressed genes was generated. The top five transcription factors identified were Sp1 Transcription Factor (*SP1*), Spi‐1 Proto‐Oncogene (*SPI1*), CCCTC‐Binding Factor (*CTCF*), Cut Like Homeobox 1 (*CUX1*), and Androgen Receptor (*AR*) (Figure 11C).

Finally, we investigated protein–chemical interactions using the Comparative Toxicogenomics Database (CTD) to identify potential therapeutic targets based on our co‐expressed gene network. The top five medications identified, excluding dangerous compounds, were valproic acid, cyclosporine, K 7174, JQ1, and estradiol (Figure 11D). These medications hold promise as innovative therapies for EC and warrant further investigation.

### 3.8 Enrichment Analysis of Gelsolin Superfamily Genes in EC

To better understand the biological roles of gelsolin superfamily genes in endometrial cancer (EC), we identified 43 genes strongly correlated with seven key members of this gene family (Figure 10B). Additionally, we performed enrichment analyses of these genes in terms of Gene Ontology (GO) and Kyoto Encyclopedia of Genes and Genomes (KEGG) functions using the Enrichr database.

**FIGURE 12:**
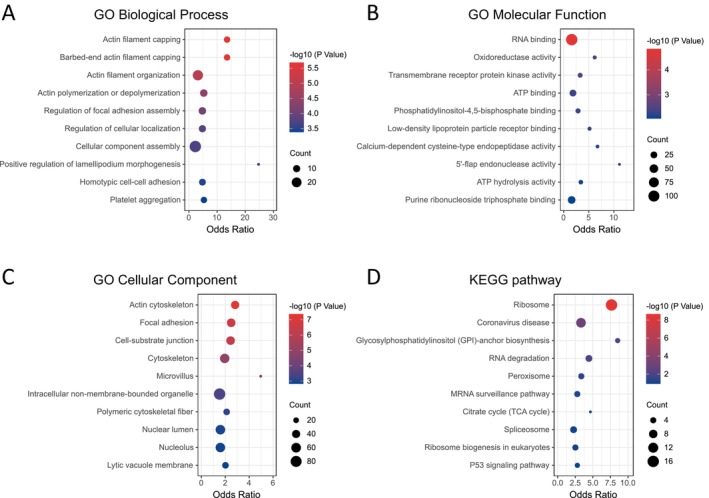
Enrichment of Gelsolin superfamily genes in UCEC (enrichr). (A) Bubble chart of GO enrichment in biological process (BP) terms. (B) Bubble chart of molecular function (MF) terms; (C) Bubble chart of cellular component (CC) terms. (D) Bubble chart of Kyoto Encyclopedia of Genes and Genomes (KEGG)‐enriched terms.

The results revealed significant enrichment of biological processes, including actin filament capping, regulation of focal adhesion assembly, cellular component assembly, and positive regulation of lamellipodium morphogenesis (Figure 12A). In terms of molecular function, the enriched categories were RNA binding, oxidoreductase activity, transmembrane receptor protein kinase activity, and ATP binding (Figure 12B). Significant cellular component enrichments included the actin cytoskeleton, focal adhesion, cell–substrate junction, microvillus, and nuclear lumen (Figure 12C). KEGG pathway analysis identified significant enrichment pathways, such as the ribosome, RNA degradation, peroxisome, mRNA surveillance pathway, citrate cycle, and p53 signaling pathway (Figure 12D).

**FIGURE 13:**
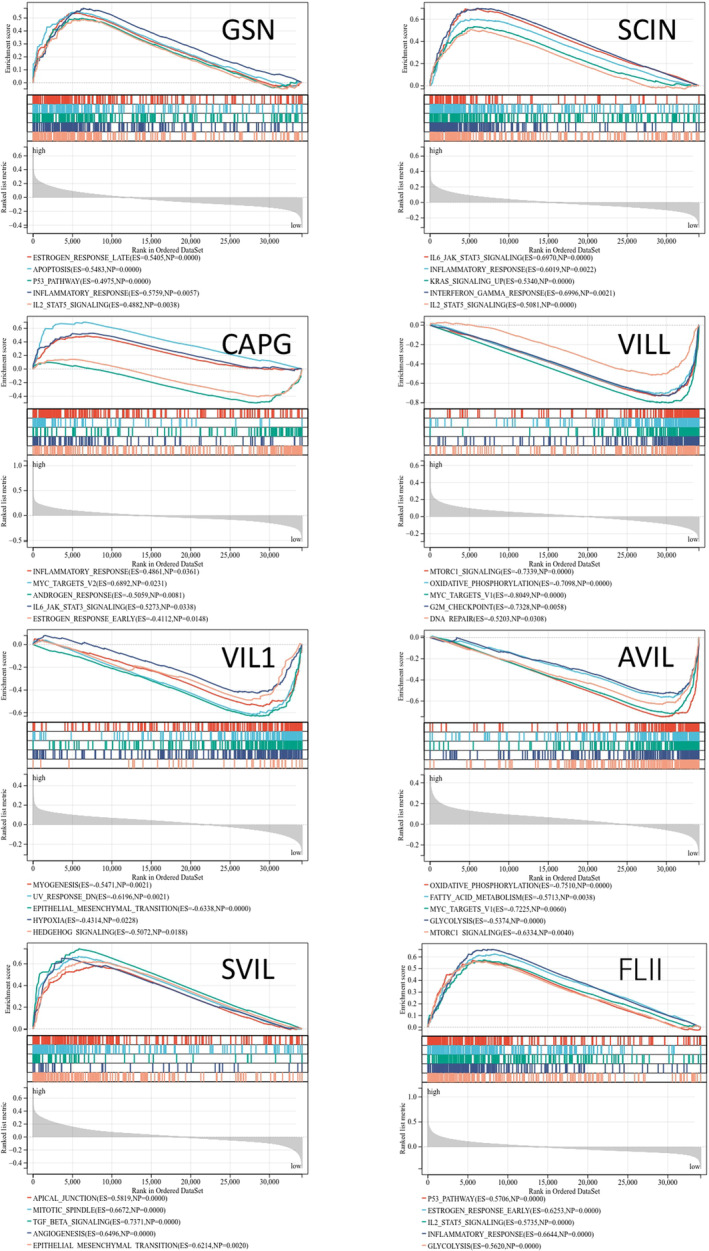
Significantly enriched gene sets in UCEC cohort from GSEA, Wiki pathway and Panther pathway annotations of Gelsolin superfamily genes. ES, Enrichment Score; NP, Normalized *p* value.

Gene Set Enrichment Analysis (GSEA) of the co‐expression genes in the TCGA‐UCEC cohort revealed significant enrichment of gene sets associated with estrogen response, MYC targets v2, epithelial–mesenchymal transition, TGF‐beta signaling, MTORC1 signaling, oxidative phosphorylation, inflammatory response, and IL6‐JAK‐STAT3 signaling (Figure 13). These results indicate that the gelsolin superfamily genes play a key role in tumor progression, metabolism, and immune regulation in a wide range of pathways.

### 3.9 Immunological Correlation of Gelsolin Superfamily Members in EC

**FIGURE 14:**
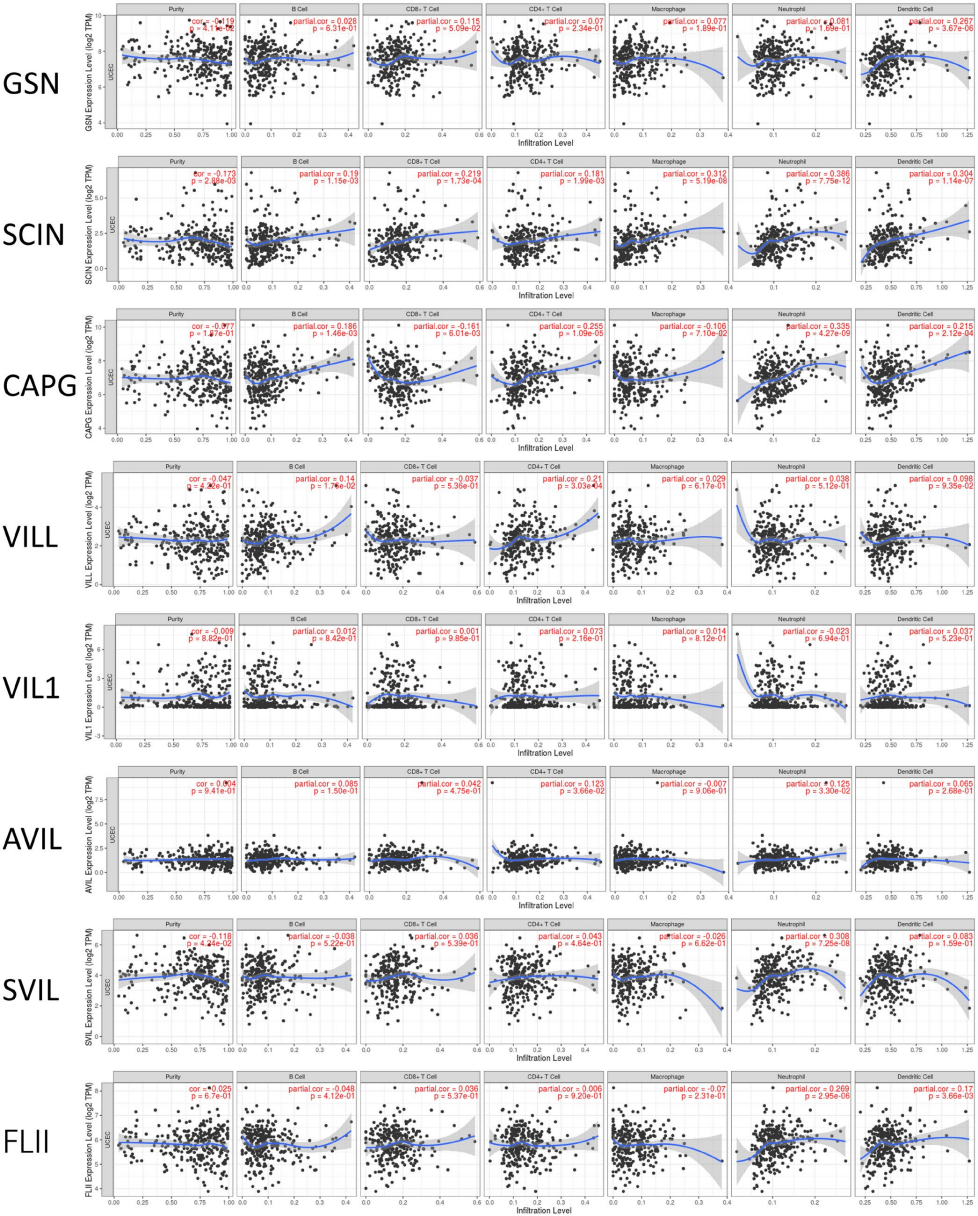
Relationship between differentially expressed Gelsolin superfamily genes and immune cell infiltration. The immune cells we analyzed included B cells, CD8+ T cells, CD4+ T cells, macrophages, neutrophils, and dendritic cells.

Our enrichment analysis revealed that gelsolin superfamily members play a role in immune responses, prompting an investigation into their correlation with immune cell infiltration using the TIMER database. *GSN* transcription levels positively correlated with dendritic cells (DCs) infiltration. *SCIN* and *CAPG* expression showed positive correlations with the infiltration of immune cell types, including B cells, CD8+ T cells, CD4+ T cells, neutrophils, and DCs. *VILL*, *AVIL*, *SVIL*, and *FLII* expression levels positively correlated with infiltration of B cells, CD4+ T cells, neutrophils, and DCs. *VIL1* expression did not show significant associations with immune cell infiltration (Figure 14).

**FIGURE 15:**
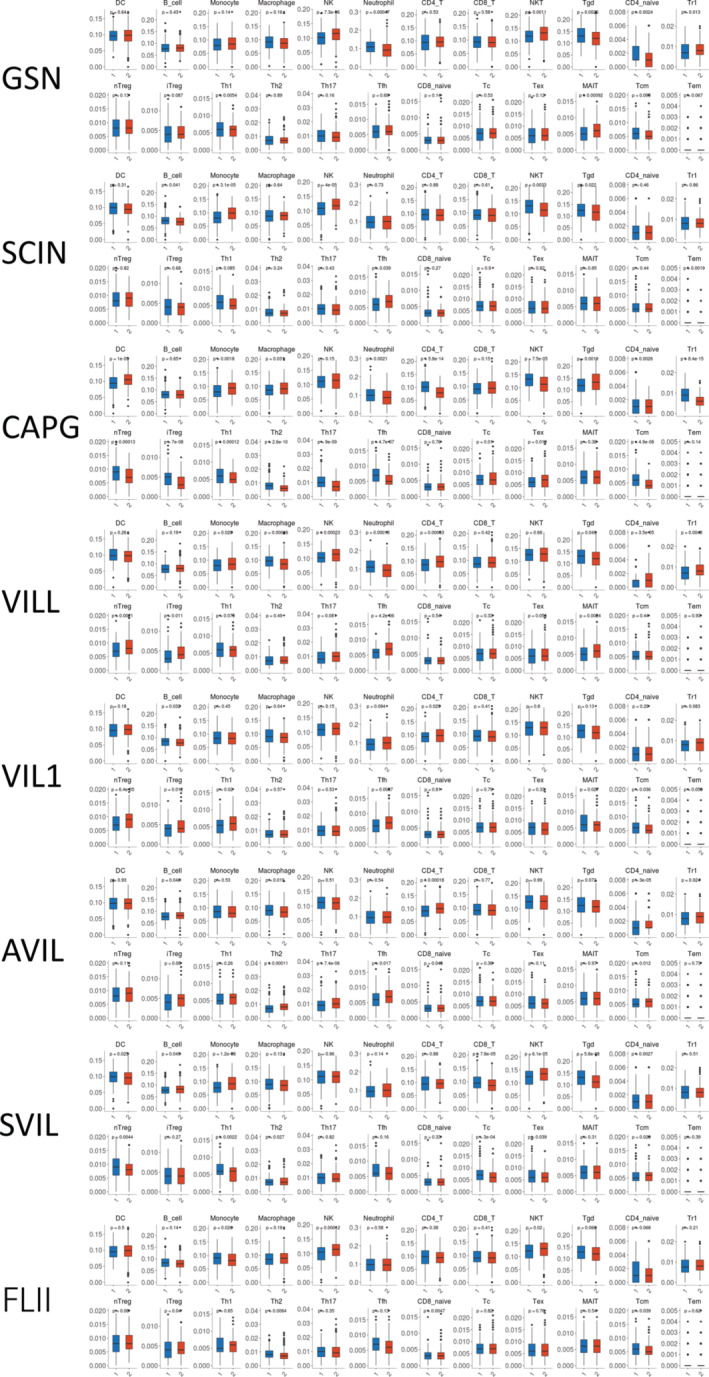
Correlations of Gelsolin superfamily genes expression with immune cell subtypes infiltration level in UCEC; blue/red box represent low/high gene expression group.

Furthermore, a quantification algorithm from the ImmuCellAI database was used to analyze the connections between gelsolin superfamily expressions and various immune cell subtypes. It was observed that monocytes, natural killer T cells (NKT), naïve CD4+ T cells, follicular helper T cells (Tfh), and central memory T cells (Tcm) exhibited the strongest relationships with gelsolin superfamily members. Additionally, moderate correlations were observed with macrophages, natural killer cells (NK), CD4+ T cells, gamma delta T cells, type 1 regulatory T cells, natural regulatory T cells, induced regulatory T cells, type 1 helper T cells (Th1), and type 2 helper T cells (Th2) (Figure 15). Among the eight gelsolin superfamily genes, *CAPG*, *VILL*, and *SVIL* showed high associations with immune cell infiltration, highlighting their crucial roles in the tumor microenvironment (TME) and immunological function.

### 3.10 Correlation Between Gelsolin Superfamily Genes Expression and Immune Marker Genes

To elucidate the immunological relevance of gelsolin superfamily members, we examined their relationship with immune cell markers in EC. This analysis encompassed immune marker genes associated with B cells, NK cells, T cells, Th1/Th2/Th17 cells, Tfh cells, regulatory T cells (Treg), tumor‐associated macrophages (TAM), M1/M2 macrophages, neutrophils, basophils, eosinophils, myeloid‐derived suppressor cells (MDSC), dendritic cells (DC), mast cells, endothelial cells, and exhausted T cells (Tex) in EC. We utilized TIMER to adjust the correlation based on purity or age (Table S1).

**FIGURE 16:**
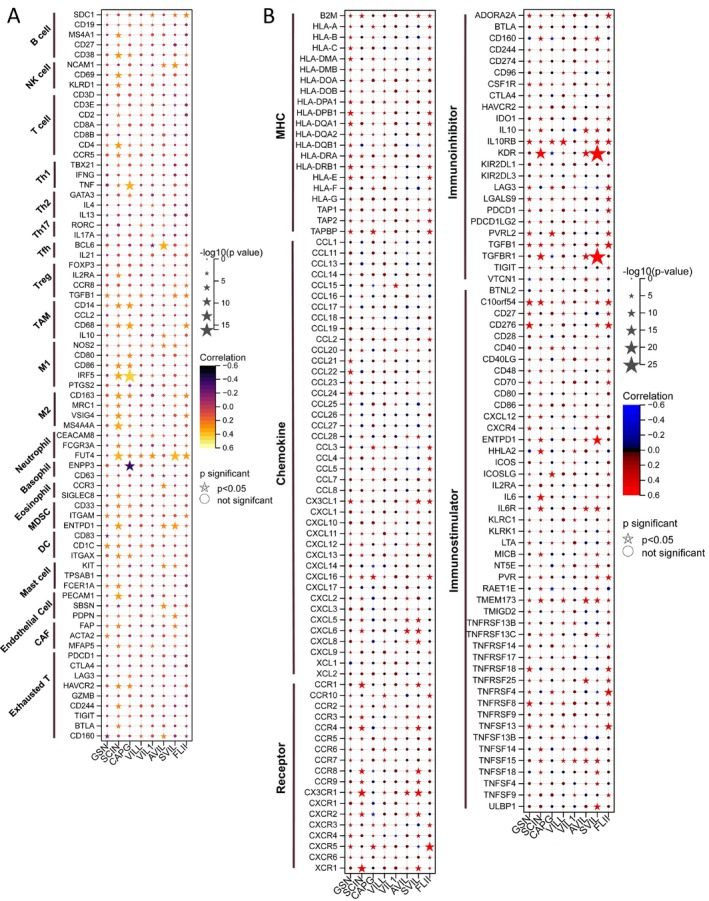
The effect of Gelsolin superfamily genes on immunological status in UCEC. (A) Correlation between Gelsolin superfamily genes and marker genes of immune cells. (B) Correlation between Gelsolin superfamily genes and 122 immunomodulators (chemokines, receptors, MHC, and immunostimulators).

Our results indicate significant correlations between the expression of *SDC1* in B cells, *TGFB1* in Treg cells, *CD68* in TAMs; *NOS2* and *IRF5* in M1 macrophages; *FUT4* in neutrophils; *ITGAM* and *ENTPD1* in MDSCs; *CD83* in DC; *KIT* in mast cells; *PDPN* in endothelial cells; *MFAP5* in cancer‐associated fibroblasts (CAF); and *PD1*, *BTLA*, and *CD160* in Tex cells with most gelsolin superfamily members in ECs (Figure 16A).

Furthermore, our co‐expression analyses aimed to identify potential immunotherapy candidates among EC patients based on gelsolin superfamily gene expression. We observed a strong association between the gelsolin superfamily and a plethora of immunomodulators in EC (Figure 16B). Specifically, gelsolin superfamily members exhibited robust co‐expression with several immune inhibitor genes, including *CD274*, *CSF1R*, *IL10RB*, *KDR*, *LAG3*, *LGALS9*, *PDCD1*, *PDCD1LG2*, *TGFB1*, *TGFBR1*, and *TIGIT*. Interestingly, we did not observe similar strong correlations with other groups of immunomodulator genes, such as major histocompatibility complex (MHC), chemokine, and immune‐stimulator genes. These findings underscore the complex interplay between the gelsolin superfamily genes and immune‐related markers in EC, highlighting their potential utility in predicting immunotherapy responses.

**FIGURE 17:**
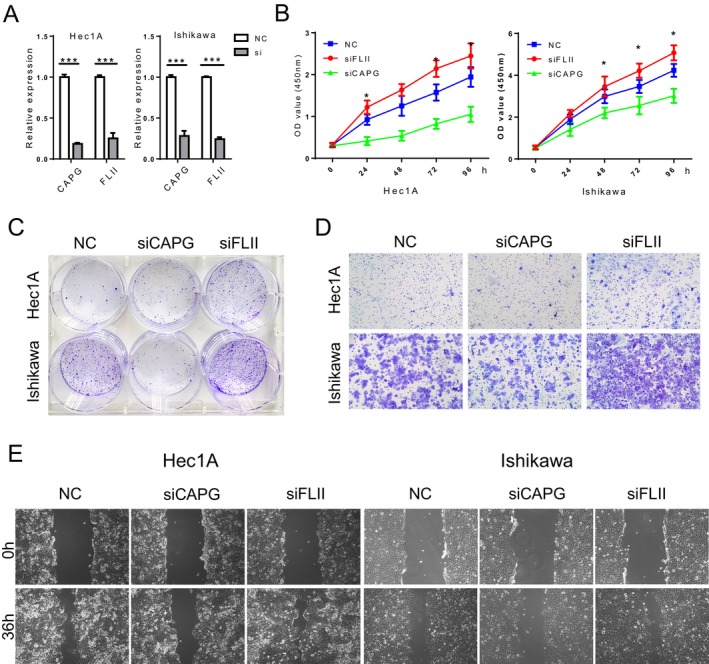
Correlation between CAPG and FLII expression and proliferation and migration of EC in vitro. (A) RT‐PCR demonstrate overexpression of CAPG and FLII in Hec1A cells and Ishikawa cells. (B, C) The proliferation was examined by CCK‐8 and colony‐forming assays. (D, E) The migration and invasion were examined by wound healing and transwell assays. (F) Related DEGs′ RNA expression was analyzed using RT‐PCR. *P < 0.05, **P < 0.01, ***P < 0.001.

### 3.11 Correlation Between *CAPG* and *FLII* Expression and the Proliferation and Migration of EC Cells In Vitro

Based on the previous findings, we propose a hypothesis stating that *CAPG* and *FLII* expression may influence the aggressive characteristics exhibited by endometrial cancer cells. Through the use of a siRNA transfection system, we successfully knocked down *CAPG* and *FLII* expression in Hec1A and Ishikawa cells. The levels of *CAPG* and *FLII* gene expression were evaluated in the modified cell lines through RT‐PCR analysis (Figure 17A).

CCK‐8 assays showed that FLII knockdown enhanced the proliferation rate of Hec1A and Ishikawa cells, while suppressing *CAPG* significantly inhibited cell growth (Figure 17B). This was further supported by colony formation experiments, where knockdown of *CAPG* decreased cell proliferation while knockdown of *FLII* had the opposite effect (Figure 17C).

In contrast to control cells, Transwell assays revealed that EC cells with reduced *FLII* expression exhibited enhanced migration, while cells with decreased *CAPG* expression displayed reduced migration (Figure 17D). Similarly, wound‐healing assays demonstrated that alterations in FLII knockdown affected the migratory speed of cells (Figure 17E). These findings suggest that high *CAPG* and low *FLII* expression levels contribute to enhanced migration capacity in EC cells.

## 4 Discussion

Endometrial cancer (EC) presents a unique challenge among prevalent cancers, with its incidence and associated mortality on an upward trajectory in developed nations [4]. Traditional medications have limited effectiveness in treating advanced EC [23]. The rising prevalence of EC is largely attributed to increased exposure to estrogens, both endogenous and exogenous. Key factors contributing to this hormonal influence include obesity, diabetes, advanced age, late‐onset menopause, nulliparity, and the utilization of tamoxifen [2, 24]. Obesity and diabetes, often components of metabolic syndrome, are also established risk factors for EC [25]. The increase in obesity rates, a significant risk factor for EC, has paralleled the rise in EC incidence [4]. Interestingly, while obesity significantly increases EC risk, it does not appear to affect EC‐specific mortality [26]. The correlation between diabetes and EC remains controversial. Only a limited number of studies have established an independent connection between diabetes mellitus and mortality rates specific to EC [27].

Our analysis of the TCGA‐UCEC cohort aligns with previous findings, showing that diabetes, hypertension, and body mass index (BMI) are not independent prognostic factors for EC. Interestingly, we observed a negative correlation between the expression of specific gelsolin superfamily members (*CAPG*, *SVIL*, and *FLII*) and BMI. Furthermore, gelsolin superfamily members, particularly *VILL* and *AVIL*, are significantly enriched in metabolic pathways including the citrate cycle, ATP hydrolysis activity, oxidative phosphorylation, and fatty acid metabolism. This finding suggests a potential role for gelsolin superfamily members in processes related to metabolic syndrome, warranting further investigation.

Age remains a significant risk factor for EC, as it does for many cancers [3]. The majority of EC cases occur in women over 50, with a median age of 63 at diagnosis. Postmenopause are most commonly affected, and late menopause has been linked to an increased risk of EC [28]. Our study found that age is an independent prognostic factor for EC, whereas menopausal status is not. Estrogen and selective estrogen receptor modulators have been shown to enhance EC susceptibility [7, 29]. Both Estrogen and tamoxifen can influence cellular signaling pathways that promote cell growth and proliferation [30]. Our results demonstrated that certain gelsolin superfamily members, particularly *GSN*, *CAPG*, and *FLII*, were enriched in estrogen/androgen response, suggesting they may play a role in regulating these signaling pathways. However, in our analysis, neither hormone therapy nor tamoxifen use was significantly associated with overall EC patient survival. This may be due to the small number of TCGA‐UCEC cohort patients who used hormone therapy (19 out of 541 patients) or tamoxifen (8 out of 541 patients).

The gelsolin superfamily genes play diverse and often controversial roles in various cancers [31]. For instance, GSN expression is downregulated in human bladder cancer, where its overexpression can actually reduce tumorigenicity [32]. Conversely, GSN is upregulated in metastatic hepatocellular carcinoma tissues, promoting cell proliferation [33, 34]. SCIN enhances the progression of gastric cancer by modulating STAT3 and NF‐Κb signaling pathways. Suppression of SCIN inhibits proliferation and induces cell cycle arrest in human prostate cancer cells [35, 36]. High CAPG expression correlates with shorter relapse‐free survival time and paclitaxel resistance in breast cancer [37]. Villin expression is frequently lost in poorly differentiated colorectal cancer [38]. AVIL overexpression promotes glioma cell proliferation and migration by regulating FOXM1 and LIN28B [39]. SVIL promotes EMT and metastasis in HCC through the RhoA/ROCK‐ERK/p38 pathway [40] and modulates androgen receptor activity [41]. FLII homolog inhibits androgen receptor signaling, slowing prostate cancer development [42], while FLII itself inhibits selective autophagy and promotes breast cancer growth by blocking p62‐mediated recognition of LC3 [43]. Despite their established impact on patient survival in various cancers, the predictive influence of the gelsolin superfamily genes on EC remains understudied. In EC, *SCIN* and *CAPG* exhibited significantly elevated expression levels compared to normal tissues, whereas *GSN*, *VILL*, and *SVIL* displayed markedly reduced expression. Consequently, clinicopathological characteristics and the expression levels of *GSN*, *SCIN*, *CAPG*, *VILL*, *VIL1*, *SVIL*, and *FLII* were found to be closely associated with tumor stages in EC. Cox analysis revealed that high expressions of *CAPG*, *AVIL*, and *SVIL* and low expressions of *GSN* and *FLII* were unfavorable predictors for EC. The low expression of *CAPG*, *AVIL*, and *SVIL*, together with the high expression of *GSN*, *VILL*, and *FLII*, were found to be significantly associated with poor overall survival in endometrial cancer patients. GSEA was conducted on the gelsolin superfamily co‐expression genes to explore the mechanisms underlying their impact on EC prognosis. The analysis revealed that *GSN* and *FLII* were positively enriched in gene sets associated with apoptosis, P53 pathways, inflammatory response, and IL2‐STAT5 signaling. VILL showed negative enrichment in gene sets associated with MTORC1 signaling, MYC targets V1, G2M checkpoint, and DNA repair. *CAPG* and *FLII* exhibited positive enrichment in gene sets like MYC targets, IL6‐JAK‐STAT3 signaling, TGF‐beta signaling, angiogenesis, and epithelial–mesenchymal transition.

The differential expression of gelsolin superfamily genes in EC may influence various aspects of tumor malignancy. Their involvement in the TME could contribute to differences in survival. Previous studies have suggested that the gelsolin superfamily genes function as a regulator of the immune system and inflammation processing. Extracellular GSN plays a role in identifying bacterial wall compounds and immune responses [44], while intracellular GSN is crucial for macrophage recruitment and motility [45]. Silencing SCIN restores normal cell shape and reduces resistance to CTL death [46]. CAPG is involved in macrophage activities such as receptor‐mediated ruffling, phagocytosis, and vesicle transport, with a potential role in TAM polarization in glioma [47]. SVIL variations produce neoepitopes that can stimulate CD8+ T‐cell responses [48]. FLII interacts with crucial inflammatory pathways such as the NLRP3 inflammasome and MyD88‐TLR4 signaling pathway, thus playing a role in innate immunity across various species [49]. In addition, FLII has the potential to overcome enzalutamide resistance by inhibiting the YBX1/PD‐L1 pathway and enhancing T‐cell responses in TME [50].

Our findings reveal a positive correlation between the presence of anticancer immune cells, including monocytes, NK cells, NKT cells, Th1 cells, Tfh cells, and mucosal‐associated invariant T cells (MAIT), and the expression of *GSN*, *VILL*, and *FLII*. Conversely, *CAPG*, *AVIL*, and *SVIL* expression negatively correlated with anticancer immune cells and positively correlated with immunosuppressive cells like TAMs, Tregs, Th2 cells, MDSCs, and cancer‐associated fibroblasts (CAFs). Additionally, gelsolin superfamily members show strong co‐expression with immune‐inhibitory genes, including*CD274*, *CSF1R*, *IL10RB*, *KDR*, *LAG3*, *LGALS9*, *PDCD1*, *PDCD1LG2*, *TGFB1*, *TGFBR1*, and *TIGIT*. These observations indicate that the gelsolin superfamily genes may modulate the balance between pro‐tumor and antitumor immune responses, resulting in an immunosuppressive TME and T cell exhaustion. The immune‐modulating effects of the gelsolin superfamily members underscore their potential as targets for enhancing cancer immunotherapy efficacy.

## 5 Conclusions

We have identified the expression levels of *GSN*, *CAPG*, *AVIL*, *SVIL*, and *FLII* as independent survival risk factors in endometrial cancer (EC) patients. The phenotypes related to the gelsolin superfamily members play crucial roles in tumor progression, metabolism, and immune regulation. Moreover, our analysis revealed strong correlations between gelsolin superfamily members and the levels of monocytes, natural killer T cells, naive CD4+ T cells, follicular helper T cells, and central memory T cells in EC. These findings highlight the potential of gelsolin superfamily members as prognostic biomarkers and immunotherapy targets in EC, opening new avenues for personalized treatment strategies and improved patient outcomes.

## Author Contributions

**Senwei Jiang:** conceptualization (lead), data curation (equal), funding acquisition (equal), validation (lead), visualization (equal), writing – original draft (lead). **Minjuan Ye:** data curation (equal), formal analysis (equal), methodology (equal), writing – review and editing (lead). **Jing Wan:** methodology (equal). **Qingjian Ye:** funding acquisition (equal), visualization (supporting). **Suli Qiu:** validation (supporting). **Yuebo Yang:** conceptualization (supporting), project administration (equal), supervision (supporting), writing – review and editing (equal). **Xiaomao Li:** funding acquisition (equal), project administration (equal), supervision (lead).

## Ethics Statement

All data collection in studies involving human participants was in accordance with the ethical standards of TCGA and HPA subject protection and data access policies.

## Consent

The authors have nothing to report.

## Conflicts of Interest

The authors declare no conflicts of interest.

## Supporting information


**Table S1.** Correlation analysis between gelsolin family and relate genes and markers of immune cells in TIMER.
**Table S2.** siRNA target sequences and PCR primers.

## Data Availability

The datasets analyzed during the current study are available from the corresponding author on reasonable request.
