# Supplementary material for: Significance of Gelsolin Superfamily Genes in Diagnosis, Prognosis and Immune Microenvironment Regulation for Endometrial Cancer
Source: Cancer Med. 2025 Feb 18;14(4):e70584. doi: 10.1002/cam4.70584 (PMC11834165; doi:10.1002/cam4.70584)
Supplement: Supplementary file 1 — Table S1. Correlation analysis between gelsolin family and relate genes and markers of immune cells in TIMER. Table S2. siRNA target sequences and PCR primers. [file CAM4-14-e70584-s001.docx]

| **Table S1 Correlation analysis between gelsolin family and relate genes and markers of immune cells in TIMER.** | | | | | | | | | | | | | | | | | | | | | | | | | | | | | | | | | | | | |
| --- | --- | --- | --- | --- | --- | --- | --- | --- | --- | --- | --- | --- | --- | --- | --- | --- | --- | --- | --- | --- | --- | --- | --- | --- | --- | --- | --- | --- | --- | --- | --- | --- | --- | --- | --- | --- |
| Description | | Gene markers | | Gene Symbol | | GSN | |  | | SCIN | |  | | CAPG | |  | | VILL | |  | | VIL1 | |  | | AVIL | |  | | SVIL | |  | | FLII | |  |
|  | |  | |  | | Cor | | P | | Cor | | P | | Cor | | P | | Cor | | P | | Cor | | P | | Cor | | P | | Cor | | P | | Cor | | P |
| B cell | | CD19 | | CD19 | | 0.022735 | | 0.698348 | | 0.115612 | | 0.048026 | | 0.175515 | | 0.00257 | | 0.145952 | | 0.012384 | | 0.12686 | | 0.029932 | | 0.062692 | | 0.284805 | | -0.04356 | | 0.457617 | | -0.06744 | | 0.249798 |
|  | | CD20 | | MS4A1 | | -0.03097 | | 0.597556 | | 0.274296 | | 1.87E-06 | | 0.128702 | | 0.02761 | | 0.135484 | | 0.020346 | | 0.09671 | | 0.098494 | | 0.087286 | | 0.136079 | | 0.019106 | | 0.744665 | | -0.08931 | | 0.127196 |
| B cell memory | | CD27 | | CD27 | | 0.034189 | | 0.559962 | | 0.128286 | | 0.028121 | | 0.083466 | | 0.154131 | | 0.135752 | | 0.020097 | | 0.038724 | | 0.509086 | | 0.037403 | | 0.523649 | | -0.10909 | | 0.062196 | | -0.04848 | | 0.40833 |
| B cell plasma | | CD38 | | CD38 | | 0.08885 | | 0.129173 | | 0.316053 | | 3.22E-08 | | 0.223702 | | 0.000113 | | 0.075022 | | 0.200376 | | 0.042629 | | 0.467288 | | 0.0084 | | 0.886159 | | 0.098475 | | 0.092472 | | 0.162735 | | 0.005233 |
|  | | CD138 | | SDC1 | | 0.129587 | | 0.026551 | | 0.065335 | | 0.264952 | | 0.17061 | | 0.003396 | | 0.039521 | | 0.5004 | | 0.21244 | | 0.00025 | | 0.043429 | | 0.458966 | | 0.213657 | | 0.000229 | | 0.264318 | | 4.50E-06 |
| Natural killer cell | | CD56 | | NCAM1 | | 0.075807 | | 0.195693 | | -0.05092 | | 0.385104 | | 0.104382 | | 0.074427 | | 0.016389 | | 0.77998 | | -0.14611 | | 0.012291 | | 0.245989 | | 2.06E-05 | | 0.304226 | | 1.09E-07 | | 0.085238 | | 0.145545 |
|  |  | CD69 | | CD69 | | 0.026617 | | 0.650017 | | 0.300987 | | 1.50E-07 | | 0.180995 | | 0.001867 | | -0.00503 | | 0.931741 | | 0.147059 | | 0.011729 | | 0.082911 | | 0.156896 | | 0.111441 | | 0.056736 | | 0.015345 | | 0.793666 |
|  |  | CD94 | | KLRD1 | | 0.059532 | | 0.309835 | | 0.261402 | | 5.78E-06 | | 0.078397 | | 0.180809 | | -0.01677 | | 0.775055 | | 0.100217 | | 0.08682 | | 0.044774 | | 0.445158 | | 0.048206 | | 0.411015 | | 0.039599 | | 0.499557 |
| T cell | | CD3 | | CD3D | | 0.041546 | | 0.47869 | | 0.110206 | | 0.059552 | | 0.112788 | | 0.05379 | | 0.124049 | | 0.033796 | | 0.053611 | | 0.360503 | | -0.04489 | | 0.443958 | | -0.16436 | | 0.004794 | | -0.12169 | | 0.037358 |
|  | | CD3E | | CD3E | | 0.058464 | | 0.318613 | | 0.172745 | | 0.003011 | | 0.085914 | | 0.142366 | | 0.142517 | | 0.014625 | | 0.080874 | | 0.167378 | | 0.013283 | | 0.82088 | | -0.12882 | | 0.027468 | | -0.09505 | | 0.104444 |
|  | | CD2 | | CD2 | | 0.000237 | | 0.996783 | | 0.189045 | | 0.001148 | | 0.125099 | | 0.032305 | | 0.129531 | | 0.026617 | | 0.061676 | | 0.292704 | | 0.000432 | | 0.994122 | | -0.11837 | | 0.04291 | | -0.10845 | | 0.063743 |
| CD8+ T cell | | CD8A | | CD8A | | 0.05504 | | 0.347827 | | 0.163332 | | 0.005068 | | 0.105662 | | 0.070923 | | 0.075423 | | 0.197974 | | 0.083669 | | 0.153131 | | 0.027162 | | 0.643342 | | -0.02776 | | 0.636089 | | -0.0308 | | 0.599578 |
|  | | CD8B | | CD8B | | 0.082746 | | 0.157727 | | -0.03335 | | 0.569678 | | -0.01041 | | 0.859217 | | 0.125116 | | 0.032282 | | -0.001 | | 0.986434 | | 0.109326 | | 0.061627 | | -0.14775 | | 0.011338 | | -0.0678 | | 0.247296 |
| CD4+ T cell | | CD4 | | CD4 | | 0.17262 | | 0.003032 | | 0.327168 | | 9.76E-09 | | 0.16587 | | 0.004415 | | 0.150628 | | 0.009821 | | 0.046813 | | 0.424682 | | 0.091794 | | 0.116914 | | -0.00224 | | 0.969601 | | 0.038408 | | 0.512547 |
|  | | CCR5 | | CCR5 | | 0.106403 | | 0.068955 | | 0.252111 | | 1.26E-05 | | 0.194845 | | 0.000799 | | 0.104792 | | 0.073291 | | 0.0345 | | 0.556403 | | 0.072412 | | 0.216524 | | 0.043831 | | 0.454811 | | 0.0837 | | 0.152978 |
| Th1 | | T-bet | | TBX21 | | 0.033418 | | 0.568859 | | 0.161002 | | 0.005742 | | 0.091747 | | 0.117103 | | 0.148179 | | 0.011098 | | 0.034547 | | 0.555858 | | 0.049361 | | 0.399887 | | -0.08368 | | 0.153065 | | -0.07648 | | 0.191721 |
|  | | IFN-γ | | IFNG | | -0.08911 | | 0.128048 | | 0.053324 | | 0.363083 | | 0.143731 | | 0.013795 | | 0.05612 | | 0.338434 | | 0.046069 | | 0.432093 | | 0.040789 | | 0.486742 | | -0.09249 | | 0.114152 | | -0.06682 | | 0.254187 |
|  | | TNF-α | | TNF | | -0.10779 | | 0.065398 | | 0.179672 | | 0.002018 | | 0.382009 | | 1.29E-11 | | 0.023265 | | 0.691679 | | 0.061819 | | 0.291582 | | 0.007442 | | 0.899064 | | 0.103998 | | 0.075507 | | 0.082537 | | 0.158784 |
| Th2 | | GATA3 | | GATA3 | | -0.0288 | | 0.623469 | | 0.090284 | | 0.123085 | | 0.214452 | | 0.000217 | | 0.050894 | | 0.385392 | | -0.07441 | | 0.204095 | | 0.034043 | | 0.561643 | | 0.072938 | | 0.213197 | | -0.02068 | | 0.724488 |
|  | | IL4 | | IL4 | | 0.006299 | | 0.914508 | | 0.017676 | | 0.763189 | | 0.021707 | | 0.711371 | | 0.151786 | | 0.009264 | | 0.153079 | | 0.008676 | | 0.076201 | | 0.193374 | | -0.064 | | 0.274877 | | -0.0813 | | 0.16516 |
|  | | IL13 | | IL13 | | -0.12454 | | 0.033098 | | 0.061506 | | 0.294035 | | -0.06202 | | 0.289996 | | -0.00654 | | 0.91122 | | -0.03813 | | 0.515572 | | 0.136244 | | 0.019646 | | -0.08826 | | 0.131754 | | -0.1042 | | 0.074952 |
| Th17 | | RORγt | | RORC | | -0.05354 | | 0.36113 | | -0.09823 | | 0.093285 | | 0.017118 | | 0.770452 | | 0.175336 | | 0.002597 | | 0.033035 | | 0.573299 | | 0.146461 | | 0.012078 | | -0.16292 | | 0.005181 | | -0.00074 | | 0.989925 |
|  | | IL17A | | IL17A | | -0.16768 | | 0.003997 | | 0.073558 | | 0.209323 | | 0.098343 | | 0.092912 | | 0.057497 | | 0.326698 | | 0.167799 | | 0.003971 | | -0.04677 | | 0.425153 | | -0.07292 | | 0.21334 | | -0.05496 | | 0.348504 |
| Tfh | | BCL6 | | BCL6 | | 0.107456 | | 0.066238 | | 0.073301 | | 0.210925 | | -0.07833 | | 0.181202 | | 0.069906 | | 0.232892 | | -0.20166 | | 0.000515 | | 0.376019 | | 2.84E-11 | | 0.104851 | | 0.073129 | | 0.149393 | | 0.010447 |
|  | | IL21 | | IL21 | | -0.02117 | | 0.718245 | | 0.082335 | | 0.159809 | | 0.088635 | | 0.130108 | | 0.038709 | | 0.509251 | | 0.104467 | | 0.074191 | | -0.01207 | | 0.837007 | | 0.100847 | | 0.084847 | | 0.108692 | | 0.063161 |
| Treg | | FOXP3 | | FOXP3 | | 0.085602 | | 0.143828 | | 0.109228 | | 0.061863 | | 0.098842 | | 0.091255 | | 0.092534 | | 0.113983 | | 0.01686 | | 0.773816 | | 0.135814 | | 0.020039 | | 0.027039 | | 0.644844 | | 0.066407 | | 0.257173 |
|  | | CD25 | | IL2RA | | 0.081157 | | 0.165892 | | 0.245079 | | 2.22E-05 | | 0.083742 | | 0.152767 | | -0.06251 | | 0.286192 | | 0.021244 | | 0.717258 | | -0.0122 | | 0.835327 | | 0.04953 | | 0.398273 | | 0.116049 | | 0.047183 |
|  | | CCR8 | | CCR8 | | 0.098916 | | 0.091012 | | 0.108885 | | 0.062689 | | 0.093519 | | 0.110167 | | -0.00763 | | 0.896575 | | 0.079672 | | 0.173798 | | 0.066849 | | 0.254012 | | 0.19246 | | 0.000929 | | 0.198196 | | 0.000645 |
|  | | TGFβ | | TGFB1 | | 0.238501 | | 3.71E-05 | | 0.183709 | | 0.001588 | | 0.16453 | | 0.00475 | | 0.207054 | | 0.00036 | | -0.00958 | | 0.870333 | | 0.152071 | | 0.009131 | | 0.258464 | | 7.41E-06 | | 0.253089 | | 1.16E-05 |
| Monocyte | | CD14 | | CD14 | | 0.18653 | | 0.001339 | | 0.289741 | | 4.49E-07 | | 0.3106 | | 5.68E-08 | | 0.030677 | | 0.600989 | | -0.03432 | | 0.55848 | | 0.021543 | | 0.713455 | | -0.0004 | | 0.994586 | | 0.166541 | | 0.004256 |
| TAM | | CCL2 | | CCL2 | | -0.0538 | | 0.35885 | | 0.136916 | | 0.019045 | | 0.014887 | | 0.799694 | | 0.072645 | | 0.215047 | | -0.03827 | | 0.514088 | | 0.038517 | | 0.511353 | | 0.007473 | | 0.898643 | | -0.04411 | | 0.45192 |
|  | | CD68 | | CD68 | | 0.178647 | | 0.002143 | | 0.268982 | | 3.00E-06 | | 0.334788 | | 4.19E-09 | | 0.03132 | | 0.593374 | | 0.053357 | | 0.362787 | | -0.00844 | | 0.885601 | | 0.118162 | | 0.043274 | | 0.28966 | | 4.52E-07 |
|  | | IL10 | | IL10 | | 0.058186 | | 0.320922 | | 0.16584 | | 0.004423 | | 0.154627 | | 0.008015 | | -0.02896 | | 0.62146 | | -0.06832 | | 0.243693 | | 0.224996 | | 0.000102 | | 0.11529 | | 0.048657 | | 0.093753 | | 0.109275 |
| M1 Macrophage | | INOS | | NOS2 | | 0.135232 | | 0.020582 | | 0.135092 | | 0.020715 | | 0.144968 | | 0.012992 | | 0.151613 | | 0.009346 | | 0.168559 | | 0.003808 | | 0.239729 | | 3.37E-05 | | 0.225932 | | 9.57E-05 | | 0.136125 | | 0.019754 |
|  |  | CD80 | | CD80 | | -0.00606 | | 0.917776 | | 0.179273 | | 0.002066 | | 0.265983 | | 3.90E-06 | | -0.01511 | | 0.796816 | | 0.076296 | | 0.192814 | | 0.084825 | | 0.14751 | | 0.112265 | | 0.054917 | | 0.140803 | | 0.01587 |
|  |  | CD86 | | CD86 | | 0.068094 | | 0.245259 | | 0.297076 | | 2.21E-07 | | 0.277855 | | 1.36E-06 | | 0.033556 | | 0.567262 | | -0.00454 | | 0.938272 | | 0.024202 | | 0.679927 | | 0.012181 | | 0.83552 | | 0.017643 | | 0.763623 |
|  | | IRF5 | | IRF5 | | -0.07234 | | 0.217001 | | 0.361398 | | 1.81E-10 | | 0.466029 | | 3.34E-17 | | 0.085153 | | 0.145946 | | 0.141584 | | 0.015292 | | 0.06452 | | 0.270973 | | 0.095701 | | 0.102074 | | 0.115382 | | 0.048476 |
|  | | COX2 | | PTGS2 | | 0.10122 | | 0.083694 | | 0.022776 | | 0.697833 | | -0.17177 | | 0.003181 | | -0.06224 | | 0.288308 | | -0.06275 | | 0.284335 | | 0.094311 | | 0.107174 | | 0.06893 | | 0.239494 | | -0.00525 | | 0.928723 |
| M2 Macrophage | | CD163 | | CD163 | | 0.108259 | | 0.064223 | | 0.323996 | | 1.38E-08 | | 0.251918 | | 1.28E-05 | | -0.04014 | | 0.493664 | | -0.10081 | | 0.084972 | | 0.055638 | | 0.342608 | | 0.199313 | | 0.0006 | | 0.268944 | | 3.01E-06 |
|  |  | CD206 | | MRC1 | | 0.181002 | | 0.001866 | | 0.241204 | | 3.01E-05 | | 0.014516 | | 0.804584 | | 0.074939 | | 0.200872 | | 0.026038 | | 0.657138 | | 0.003634 | | 0.950614 | | 0.161543 | | 0.005578 | | 0.07318 | | 0.211677 |
|  | | VSIG4 | | VSIG4 | | 0.142726 | | 0.014478 | | 0.308882 | | 6.77E-08 | | 0.197037 | | 0.000695 | | -0.02526 | | 0.666767 | | -0.04363 | | 0.456884 | | 0.02766 | | 0.637266 | | 0.103069 | | 0.07817 | | 0.245068 | | 2.22E-05 |
|  | | MS4A4A | | MS4A4A | | 0.099204 | | 0.090071 | | 0.367399 | | 8.56E-11 | | 0.175985 | | 0.002502 | | 0.025469 | | 0.664171 | | -0.01294 | | 0.825483 | | 0.011665 | | 0.842403 | | 0.073983 | | 0.206696 | | 0.095379 | | 0.103237 |
| Neutrophil | | CD66b | | CEACAM8 | | 0.03051 | | 0.602968 | | 0.034964 | | 0.551103 | | -0.14177 | | 0.015158 | | 0.152183 | | 0.00908 | | 0.19169 | | 0.000974 | | 0.129673 | | 0.026451 | | 0.02449 | | 0.676339 | | 0.041543 | | 0.478713 |
|  | | CD16 | | FCGR3A | | 0.025382 | | 0.665242 | | 0.246947 | | 1.91E-05 | | 0.160259 | | 0.005973 | | -0.06044 | | 0.302532 | | -0.0725 | | 0.215963 | | 0.052546 | | 0.370135 | | 0.053246 | | 0.363788 | | 0.153796 | | 0.008364 |
|  | | CD15 | | FUT4 | | -0.01023 | | 0.861532 | | 0.384657 | | 9.08E-12 | | 0.23364 | | 5.39E-05 | | 0.225114 | | 0.000102 | | 0.301717 | | 1.40E-07 | | 0.10096 | | 0.084494 | | 0.397975 | | 1.47E-12 | | 0.330742 | | 6.58E-09 |
| Basophil | | CD203c | | ENPP3 | | 0.110318 | | 0.05929 | | -0.01088 | | 0.852825 | | -0.39071 | | 4.01E-12 | | 0.096857 | | 0.09798 | | -0.02142 | | 0.71498 | | 0.042899 | | 0.464465 | | -0.13102 | | 0.024906 | | -0.0277 | | 0.636747 |
|  | | CD63 | | CD63 | | 0.154628 | | 0.008015 | | 0.023472 | | 0.689078 | | 0.074858 | | 0.201364 | | -0.00367 | | 0.950102 | | -0.06718 | | 0.251659 | | -0.15922 | | 0.006311 | | -0.13437 | | 0.021413 | | -0.05698 | | 0.331101 |
| Eosinophil | | CD193 | | CCR3 | | -0.02162 | | 0.71246 | | 0.069028 | | 0.238828 | | 0.037933 | | 0.517789 | | 0.028105 | | 0.631852 | | -0.02498 | | 0.670252 | | 0.278504 | | 1.28E-06 | | 0.017328 | | 0.767712 | | -0.00319 | | 0.956643 |
|  | | Siglec-8 | | SIGLEC8 | | -0.02076 | | 0.723483 | | 0.25522 | | 9.71E-06 | | 0.114853 | | 0.049522 | | 0.049235 | | 0.401095 | | 0.044663 | | 0.446289 | | 0.083363 | | 0.154641 | | 0.00668 | | 0.90935 | | 0.084693 | | 0.148145 |
| MDSC | | CD33 | | CD33 | | 0.092222 | | 0.115209 | | 0.242835 | | 2.65E-05 | | 0.217978 | | 0.00017 | | 0.094642 | | 0.105939 | | -0.03324 | | 0.570908 | | 0.134934 | | 0.020866 | | 0.047147 | | 0.421385 | | 0.086492 | | 0.13969 |
|  | | CD11b | | ITGAM | | 0.229696 | | 7.24E-05 | | 0.211152 | | 0.000273 | | 0.19463 | | 0.00081 | | 0.100366 | | 0.086351 | | 0.025606 | | 0.662478 | | 0.069959 | | 0.232536 | | 0.098125 | | 0.09364 | | 0.221139 | | 0.000135 |
|  | | CD39 | | ENTPD1 | | 0.04453 | | 0.447648 | | 0.332809 | | 5.23E-09 | | -0.04742 | | 0.418744 | | 0.097537 | | 0.095634 | | 0.0054 | | 0.926671 | | 0.274733 | | 1.80E-06 | | 0.328231 | | 8.68E-09 | | 0.163378 | | 0.005055 |
| DC | | CD83 | | CD83 | | -0.18872 | | 0.001171 | | 0.148826 | | 0.010747 | | 0.197837 | | 0.00066 | | 0.000875 | | 0.988101 | | 0.00738 | | 0.899904 | | 0.18814 | | 0.001214 | | 0.123898 | | 0.034015 | | 0.020357 | | 0.728593 |
|  | | BDCA-1 | | CD1C | | 0.275825 | | 1.63E-06 | | 0.222997 | | 0.000118 | | 0.063749 | | 0.276753 | | 0.200476 | | 0.000556 | | 0.087538 | | 0.134945 | | -0.01718 | | 0.769606 | | 0.049279 | | 0.400673 | | 0.020097 | | 0.73192 |
|  | | CD11c | | ITGAX | | 0.112939 | | 0.053468 | | 0.268543 | | 3.12E-06 | | 0.240467 | | 3.19E-05 | | 0.129178 | | 0.027037 | | 0.019684 | | 0.737224 | | 0.144499 | | 0.013291 | | 0.0444 | | 0.448969 | | 0.100749 | | 0.08515 |
| Mast cell | | CD117 | | KIT | | 0.129859 | | 0.026232 | | 0.18136 | | 0.001827 | | -0.16284 | | 0.005205 | | 0.017767 | | 0.762006 | | -0.02039 | | 0.728187 | | 0.25425 | | 1.05E-05 | | 0.21165 | | 0.000263 | | 0.069358 | | 0.236584 |
|  | | Tryptase | | TPSAB1 | | 0.067404 | | 0.25008 | | 0.049167 | | 0.401738 | | -0.03715 | | 0.526466 | | 0.097085 | | 0.097188 | | 0.035512 | | 0.54487 | | 0.070568 | | 0.228483 | | -0.03676 | | 0.530877 | | -0.04111 | | 0.483322 |
|  | | FcεRI | | FCER1A | | 0.192896 | | 0.000904 | | 0.267723 | | 3.35E-06 | | 0.027651 | | 0.637372 | | 0.164493 | | 0.004759 | | 0.021979 | | 0.707911 | | 0.001183 | | 0.98391 | | 0.051849 | | 0.376526 | | -0.08738 | | 0.135645 |
| EC | | CD31 | | PECAM1 | | 0.006715 | | 0.908881 | | 0.33429 | | 4.43E-09 | | 0.074748 | | 0.202025 | | 0.057326 | | 0.328139 | | -0.08309 | | 0.156017 | | 0.086899 | | 0.137832 | | 0.013913 | | 0.812544 | | -0.12548 | | 0.031777 |
|  | | SBSN | | SBSN | | 0.05155 | | 0.379288 | | -0.1633 | | 0.005076 | | -0.03076 | | 0.600055 | | -0.04636 | | 0.429216 | | -0.02269 | | 0.698962 | | 0.283091 | | 8.39E-07 | | 0.084133 | | 0.150853 | | 0.096968 | | 0.097592 |
|  | | Podoplanin | | PDPN | | 0.123804 | | 0.034152 | | 0.15852 | | 0.006547 | | 0.121273 | | 0.03802 | | 0.107694 | | 0.065635 | | 0.119704 | | 0.040598 | | 0.163907 | | 0.004913 | | 0.262867 | | 5.10E-06 | | 0.035192 | | 0.54851 |
| CAF | | FAP | | FAP | | 0.164305 | | 0.004808 | | 0.256137 | | 9.00E-06 | | 0.047768 | | 0.415283 | | 0.025165 | | 0.667935 | | -0.06451 | | 0.271079 | | 0.063501 | | 0.278627 | | 0.239056 | | 3.56E-05 | | 0.013829 | | 0.813657 |
|  | | α‐SMA | | ACTA2 | | 0.229917 | | 7.13E-05 | | 0.077036 | | 0.188524 | | -0.06107 | | 0.297452 | | 0.129395 | | 0.026778 | | -0.05221 | | 0.373253 | | 0.082285 | | 0.160067 | | 0.178111 | | 0.002212 | | 0.049671 | | 0.396926 |
|  | | MFAP5 | | MFAP5 | | 0.012123 | | 0.836295 | | 0.209773 | | 0.0003 | | 0.159999 | | 0.006056 | | 0.033383 | | 0.569256 | | 0.174468 | | 0.002729 | | 0.051981 | | 0.375313 | | 0.152185 | | 0.009079 | | 0.042692 | | 0.466626 |
| T cell exhaustion marker | | PD-1 | | PDCD1 | | -0.0068 | | 0.907706 | | 0.032272 | | 0.582187 | | 0.054278 | | 0.35455 | | 0.093149 | | 0.111586 | | 0.000702 | | 0.990452 | | 0.089264 | | 0.127392 | | -0.16741 | | 0.004058 | | -0.06332 | | 0.279983 |
|  |  | CTLA4 | | CTLA4 | | -0.03187 | | 0.586861 | | 0.050017 | | 0.393644 | | -0.02838 | | 0.628539 | | 0.043011 | | 0.463302 | | 0.001365 | | 0.98144 | | 0.06574 | | 0.261993 | | -0.15012 | | 0.010075 | | -0.09206 | | 0.115865 |
|  | | LAG3 | | LAG3 | | -0.05598 | | 0.339661 | | 0.028077 | | 0.632199 | | 0.195627 | | 0.00076 | | 0.04911 | | 0.40229 | | -0.01841 | | 0.753603 | | 0.080332 | | 0.170247 | | -0.00569 | | 0.922714 | | 0.001275 | | 0.982662 |
|  | | TIM-3 | | HAVCR2 | | 0.08609 | | 0.141545 | | 0.273092 | | 2.08E-06 | | 0.261324 | | 5.81E-06 | | 0.021995 | | 0.707709 | | -0.00341 | | 0.95359 | | 0.027133 | | 0.643692 | | 0.017964 | | 0.759454 | | 0.102302 | | 0.080423 |
|  | | GZMB | | GZMB | | -0.01594 | | 0.785809 | | 0.052022 | | 0.374935 | | 0.086113 | | 0.141441 | | -0.02942 | | 0.615963 | | -0.03946 | | 0.501054 | | -0.01115 | | 0.849291 | | -0.11498 | | 0.049268 | | -0.07929 | | 0.175862 |
|  | | 2B4 | | CD244 | | 0.036977 | | 0.528401 | | 0.263958 | | 4.64E-06 | | 0.126588 | | 0.030289 | | 0.100021 | | 0.087442 | | 0.09262 | | 0.113645 | | 0.060021 | | 0.305869 | | -0.06399 | | 0.274946 | | -0.05287 | | 0.367205 |
|  | | TIGIT | | TIGIT | | 0.010301 | | 0.860629 | | 0.143306 | | 0.014081 | | 0.138884 | | 0.017374 | | 0.039219 | | 0.503684 | | 0.044192 | | 0.451102 | | -0.02425 | | 0.679269 | | -0.02268 | | 0.698995 | | 0.010485 | | 0.858168 |
|  | | BTLA | | BTLA | | -0.02408 | | 0.681485 | | 0.229438 | | 7.38E-05 | | 0.120984 | | 0.038483 | | 0.140098 | | 0.016409 | | 0.055293 | | 0.345609 | | 0.092164 | | 0.115443 | | -0.05164 | | 0.3785 | | -0.13227 | | 0.023553 |
|  | CD160 | | CD160 | | -0.21859 | | 0.000162 | | 0.073407 | | 0.21026 | | -0.03646 | | 0.534147 | | 0.158407 | | 0.006586 | | 0.095971 | | 0.101105 | | 0.254115 | | 1.06E-05 | | -0.08549 | | 0.144374 | | -0.14604 | | 0.012328 | |

| **Table S2. siRNA target sequences and PCR primers.** | | |
| --- | --- | --- |
|  | sense（5'-3'） | antisense（5'-3'） |
| **siRNA target sequences** | |  |
| siCAPG | GCATTTCACAAGACCTCCACA |  |
| siFLII | GCTGCCACAGATCAACTACAA |  |
| **PCR-Primers** | |  |
| CAPG | GGGGACTCCTACCTAGTGCTG | CACCACCTTCCTGGTACTTGA |
| FLII | CTGCAATGACCTGACACGG | GGTGAGCTGATTTCGGGACAG |
| MYC | GGCTCCTGGCAAAAGGTCA | CTGCGTAGTTGTGCTGATGT |
| IL6 | ACTCACCTCTTCAGAACGAATTG | CCATCTTTGGAAGGTTCAGGTTG |
| STAT3 | CAGCAGCTTGACACACGGTA | AAACACCAAAGTGGCATGTGA |
| GAPDH | CTCCTCCTGTTCGACAGTCAGC | CCCAATACGACCAAATCCGTT |
